# Intergenerational genomic DNA methylation patterns in mouse hybrid strains

**DOI:** 10.1186/gb-2014-15-5-r68

**Published:** 2014-04-30

**Authors:** Luz D Orozco, Liudmilla Rubbi, Lisa J Martin, Fang Fang, Farhad Hormozdiari, Nam Che, Andrew D Smith, Aldons J Lusis, Matteo Pellegrini

**Affiliations:** 1 Department of Molecular, Cell and Developmental Biology, University of California Los Angeles, Los Angeles, CA 90095, USA; 2 Department of Medicine, University of California Los Angeles, Los Angeles, CA 90095, USA; 3 Molecular and Computational Biology, University of Southern California, Los Angeles, CA 90089, USA; 4 Department of Computer Science, University of California Los Angeles, Los Angeles, CA 90095, USA

## Abstract

**Background:**

DNA methylation is a contributing factor to both rare and common human diseases, and plays a major role in development and gene silencing. While the variation of DNA methylation among individuals has been partially characterized, the degree to which methylation patterns are preserved across generations is still poorly understood. To determine the extent of methylation differences between two generations of mice we examined DNA methylation patterns in the livers of eight parental and F1 mice from C57BL/6J and DBA/2J mouse strains using bisulfite sequencing.

**Results:**

We find a large proportion of reproducible methylation differences between C57BL/6J and DBA/2J chromosomes in CpGs, which are highly heritable between parent and F1 mice. We also find sex differences in methylation levels in 396 genes, and 11% of these are differentially expressed between females and males. Using a recently developed approach to identify allelically methylated regions independently of genotypic differences, we identify 112 novel putative imprinted genes and microRNAs, and validate imprinting at the RNA level in 10 of these genes.

**Conclusions:**

The majority of DNA methylation differences among individuals are associated with genetic differences, and a much smaller proportion of these epigenetic differences are due to sex, imprinting or stochastic intergenerational effects. Epigenetic differences can be a determining factor in heritable traits and should be considered in association studies for molecular and clinical traits, as we observed that methylation differences in the mouse model are highly heritable and can have functional consequences on molecular traits such as gene expression.

## Background

Methylation of DNA cytosine bases is essential for mammalian development and plays an important role in X-chromosome inactivation, imprinting, regulation of gene expression, silencing of repetitive elements and differentiation. Aberrant DNA methylation in early development is lethal, and in adult humans it is associated with both rare and complex diseases, such as Prader-Willi syndrome, Angelman syndrome, cancer, aging [1] and rheumatoid arthritis [2]. In mammals, DNA methylation occurs primarily on cytosines of CG dinucleotides, although high methylation in the CHG and CHH sequence contexts (where H is any base other than G) has been observed in stem cells [3] and in adult mouse frontal cortex [4]. The genome contains CG-rich regions known as CpG islands that are predominantly unmethylated and associated with transcriptional start sites. In the remainder of the genome, CpGs are generally sparse, and these CpGs tend to be highly methylated [5-7]. While the regulation of DNA methylation by numerous enzymes has been well studied, the transgenerational inheritance of DNA methylation patterns and their relationship with genetic variation is only beginning to be elucidated.

During gametogenesis and embryogenesis, epigenetic reprogramming of the cell involves global changes in epigenetic marks, and DNA methylation patterns are erased and re-established in the progeny. A small fraction of genetic loci can escape epigenetic reprogramming and thus can be stably transmitted to the next generation [8,9], and epigenetic inheritance patterns can be influenced by both environmental [10] and genetic variation [11]. Nonetheless, to date few studies have examined transgenerational inheritance of DNA methylation patterns using genome-wide approaches, and the degree to which these patterns are faithfully reproduced across generations remains to be determined. To address these questions, we examined the influence of genetics, sex and parental origin effects on intergenerational DNA methylation profiles in the mouse liver. We constructed reciprocal crosses of the widely used laboratory mouse strains C57BL/6J and DBA/2J, and examined global DNA methylation patterns in the parents, their F1 progeny and in both females and males using reduced representation bisulfite sequencing.

## Results

### Reduced representation bisulfite sequencing data

**Figure 1:**
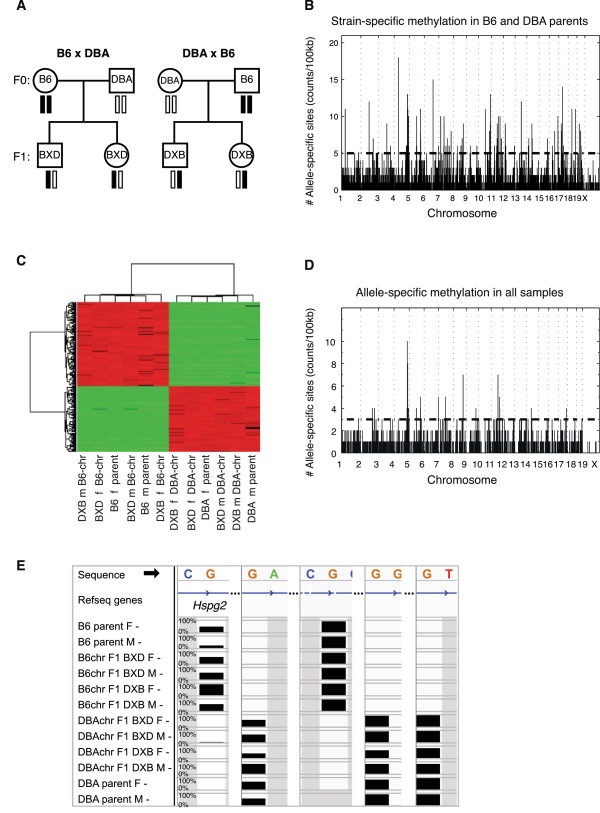
**Allele-specific methylation. (A)** Pedigree illustrating design of the mouse cross. Circles denote females and squares males. Black bars represent chromosomes derived from the C57BL/6J parents (B6) and white bars are chromosomes derived from the DBA/2J parents (DBA). **(B)** Allele-specific methylation sites between B6 and DBA female parents. The number of cytosines differentially methylated in each 100-kb bin is shown on the Y-axis and the genomic position of the bin is on the X-axis. All sites plotted are significant, and the horizontal dashed line represents the significance threshold for the 100-kb bin. **(C)** Hierarchical clustering of mice (columns) and allele-specific methylation sites (rows) for CG, CHG and CHH differentially methylated cytosines. Red indicates increased methylation and green indicates decreased methylation relative to other samples. f, female; m, male. **(D)** Allele-specific methylation sites identified using all eight mice. The number of C residues in each bin is on the Y-axis and the genomic position is on the X-axis. All sites plotted are significant, and the horizontal dashed line represents the significance threshold for each bin. **(E)** Methylation levels across all samples, showing allele-specific methylation of the gene *Hspg2.* The height of the bar represents percentage methylation from 0 to 100%. Gray represents missing data. The minus sign at the end of each sample denotes data from the minus strand.

We made reciprocal crosses of the two genetically distinct mouse inbred strains C57BL/6J (B6) and DBA/2J (DBA), and constructed reduced representation bisulfite sequencing (RRBS) libraries from liver genomic DNA of female and male B6 and DBA parents, from BXD F1 mice where the female parent is B6 and male parent is DBA, and from DXB F1 mice where the female parent is DBA and male parent is B6 (Figure 1A). We used the Illumina HiSeq to sequence the RRBS libraries and obtained, on average, 96.2 million reads (Figure S1A in Additional file 1). On average, we uniquely aligned 21.9 million reads to the mouse genome using BS Seeker [12], which corresponded to 25.6% mappability (Figure S1B in Additional file 1) and 60× average coverage (Figure S1C in Additional file 1). To estimate the sequencing error rate in our data, we compared base calls in data from B6 and DBA parents to published genotypes at known polymorphic SNPs, using publicly available data from the Wellcome Trust Sanger sequencing mouse genomes project [13]. We found that approximately 99.02% of the polymorphic reads correctly match the expected genotype in B6 (99.2%) and DBA (99.1%) mice, and approximately 0.98% of the reads did not match the expected genomic sequence. Hence, we conclude that the sequencing error rate in our RRBS data was <1% (Figure S1D in Additional file 1). Furthermore, we examined global DNA methylation levels and found no significant differences among the different samples, for which the overall cytosine methylation rate was 8.05% ± 1.72%, the CG methylation rate was 48.39 ± 7.71%, the CHG methylation rate was 0.71% ± 0.26% and the CHH methylation rate was 0.75% ± 0.26% (Figure S1E,F in Additional file 1). Global methylation levels were consistent with previous studies in mammalian somatic cells [6].

### Strain-specific methylation

To determine if individual sites in the genome were differentially methylated between the strains, we first compared individual sites between the parental B6 and DBA female mice, and between parental B6 and DBA male mice, using a binomial test [14]. We restricted our analysis to sites covered by at least 10 reads, and categorized a site as differentially methylated if the methylation level of each strain was outside of the 95% confidence interval of methylation of each other, and the absolute difference in percentage methylation between the two samples was greater than 50%, with false discovery rate (FDR) <1%. Using these criteria, we found 6,247 cytosine sites differentially methylated between B6 and DBA female parents, and 8,480 cytosines differentially methylated between B6 and DBA male parents. This corresponded to an overall allele-specific methylation rate of 0.10% (approximately 1/1,000 cytosines) in female parents, and 0.12% in males parents (1/850 cytosines). This rate was higher for CG methylation (0.56% and 0.52% in females and males) than for either CHG (0.03% and 0.06%) or CHH (0.04% and 0.03%) methylation. The genome-wide distribution of these sites is shown in Figure 1B for females and in Figure S2A in Additional file 2 for males. Although most of the cytosines in the mouse genome are non-CG, we observed that the rate of differential methylation was approximately five times higher in CGs (0.56%) relative to all cytosines (0.10%). Indeed, 75% of differentially methylated cytosines in the female parents were CGs, and 71% were in the male parents (Figure S2B,C in Additional file 2). We also observed that, of the sites that were represented in both female and male parent datasets, there was a significant overlap of 2,865 cytosines (hypergeometric *P <* 1 × 10^-16^), and the remaining were unique to either females or males (Figure S2D in Additional file 2).

**Figure 2:**
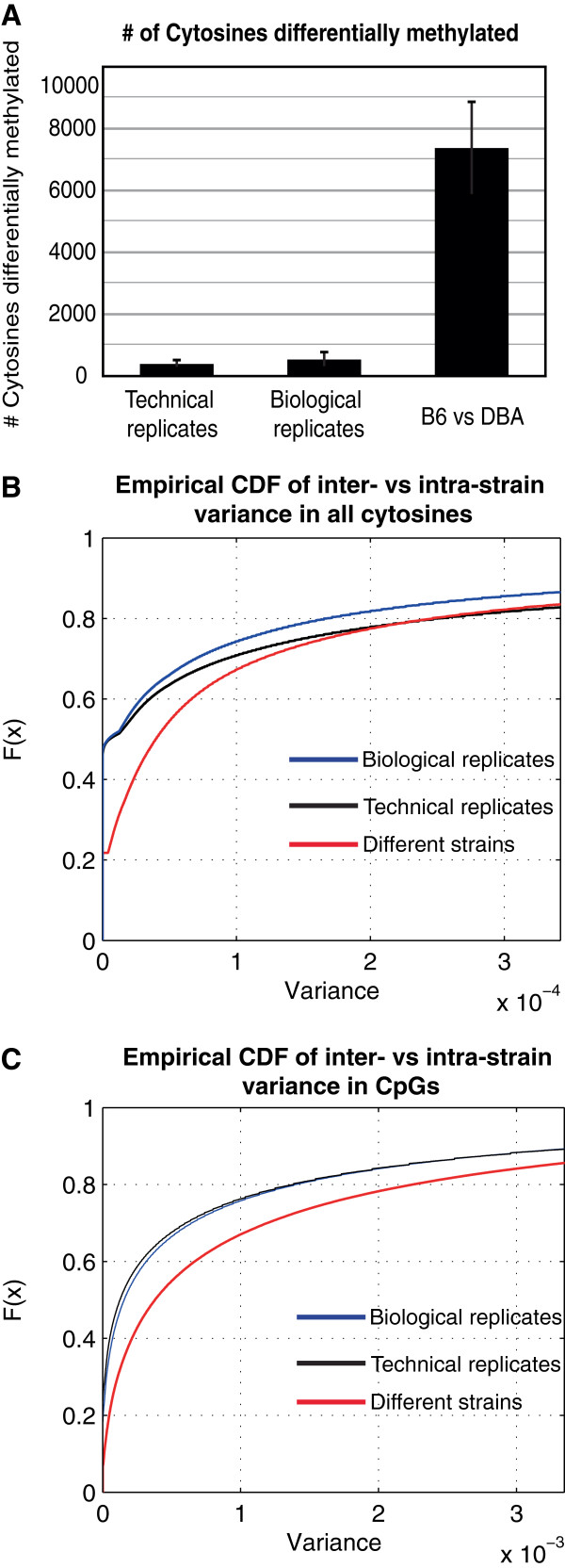
**Reproducibility of methylation levels. (A)** Differentially methylated cytosines identified in RRBS sequencing data derived from technical replicates, biological replicates, and samples from different mouse strains in B6 and DBA female or male mice. Error bars represent the standard deviation. **(B,C)** Distribution of the variance in methylation levels. The plots show the empirical cumulative distribution function (CDF) for the variance in methylation levels for all cytosines **(B)** and for CpGs **(C)**. The distribution of biological replicates is shown by the blue curve, of technical replicates by the black curve, and of different mouse strains by the red curve.

To determine the reproducibility and the degree of variation in our bisulfite sequencing data, we generated bisulfite sequencing libraries of technical replicates (different libraries from the same mouse genomic DNA sample) and biological replicates (different mice from the same strain), and looked for the presence of differentially methylated cytosines using the binomial test to compare methylation levels between pairs of samples, as described above. We found, on average, 383 differentially methylated cytosines when comparing technical replicates, and 524 differentially methylated cytosines between biological replicates (Figure 2A). In contrast, on average, we identified 7,364 differentially methylated cytosines when comparing samples from different mouse strains, which was 20-fold and 14-fold higher than the number of differentially methylated sites we identified in technical and biological replicates, respectively. Furthermore, we would expect to find a large false positive rate if variation in methylation levels between technical or biological samples was similar to the variation among different mouse strains. Therefore, to examine the degree of variation in methylation levels derived from bisulfite sequencing data, we compared the distribution of the variance in methylation levels between biological replicates (intra-strain variance) to the variance in methylation between different mouse strains (inter-strain variance) using the Kolmogorov-Smirnov (KS) test. We found that the variance in methylation levels between strains was 9-fold higher than the variance in methylation between biological replicates (KS test *P* < 1 × 10^-16^; Figure 2B) for all cytosines, and 2.4-fold higher for CpG methylation levels (KS test *P* < 1 × 10^-16^; Figure 2C).

### Allele-specific methylation

We next examined methylation levels across all samples in order to identify allele-specific methylation events that persist across inbred parents and hybrid progeny. To analyze data in the hybrid mice we first used the bisulfite sequences to identify SNPs between the two strains, and found 24,132 SNPs between B6 and DBA represented in our sequenced fragments. We used these SNPs to determine whether individual reads in the hybrid mice were derived from the B6 or DBA chromosomes, and found 127,893 cytosines in polymorphic reads tagged by SNPs. Next, we examined allele-specific methylation of reads derived from the B6 chromosomes and compared them to reads from the DBA chromosomes using a *t-*test. We selected sites where *P* < 0.05 and the difference in methylation was greater than 50% (FDR <0.05%). Here we found 2,091 differentially methylated sites, which corresponded to an allele-specific methylation rate of 5.3% for CG, 0.77% for CHG and 0.88% for CHH contexts. A clustering heatmap for differentially methylated cytosines of all contexts is shown in Figure 1C and the genome-wide distribution is shown in Figure 1D. We also observed that reads from all B6 chromosomes cluster together, separately from DBA chromosomes, even when using data from all polymorphic sites present in all samples (Figure S3 in Additional file 3). As an example, a cluster of CpGs in the gene encoding heparan sulfate proteoglycan 2 (*Hpsg2*) showed large differences in methylation between B6 and DBA chromosomes. Methylation levels across all samples are shown in Figure 1E, where the height of each bar represents the average methylation levels of 10 or more CpGs, between 0 and 100% methylation.

The number of differentially methylated sites across all samples was smaller (2,091) than the number of differentially methylated sites in the parents (6,247 and 8,480 in female and male parents, respectively), since the analysis across all samples was restricted to polymorphic reads, comprising, on average, 5% of all reads in our data. However, we observed that polymorphic fragments were much more likely to be differentially methylated than non-polymorphic fragments, since the fraction of differentially methylated CGs was 10-fold higher in polymorphic sites (5.3%) than in all CG sites in the parents (0.56% in female and 0.52% in male parents). We also observed that the number of allele-specific methylation sites across all mice were highly correlated with SNP density (Pearson’s *r* = 0.59). To further explore this correlation, we compared the distribution of differentially methylated cytosines in polymorphic versus non-polymorphic regions. We counted the number of differentially methylated cytosines in 100-kb bins across the genome, and used a KS test to compare the distribution of the counts in non-polymorphic versus polymorphic bins, containing at least one SNP. The average number of differentially methylated cytosines across all genomic bins was 17-fold higher (0.77/0.045) in polymorphic bins relative to non-polymorphic bins in female B6 versus DBA mice (KS test, *P* < 1 × 10^-16^), and 10-fold higher (0.95/0.095, KS test, *P* < 1 × 10^-16^) in male B6 versus DBA mice (Figure S2E,F in Additional file 2).

### Sex differences

**Figure 3:**
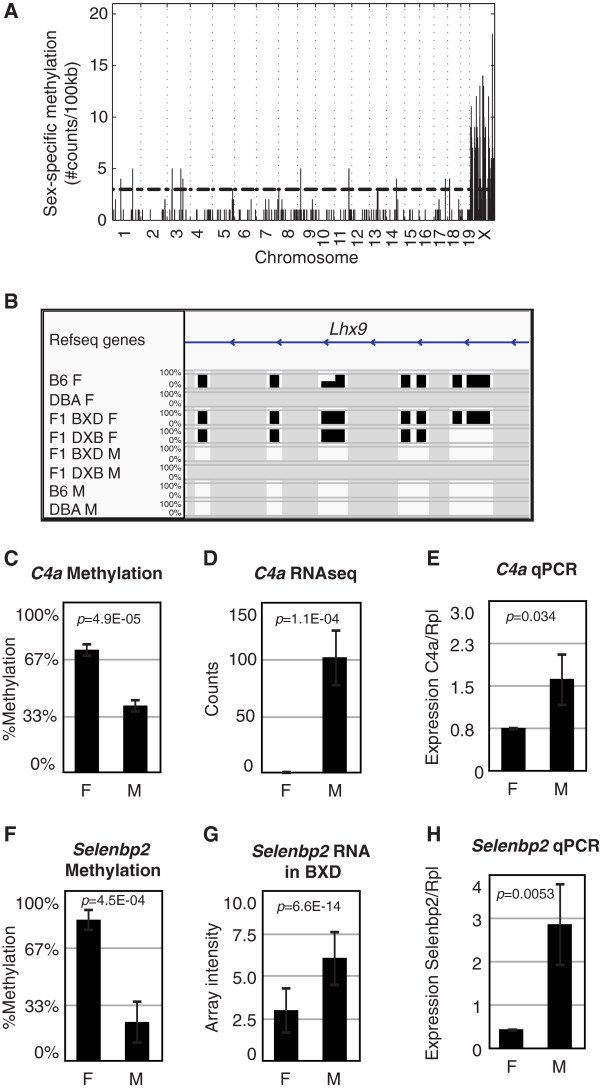
**Sex differences. (A)** Sex-specific methylation sites across the genome. The number of cytosines differentially methylated in each 100-kb bin is shown on the Y-axis and the genomic position of the bin is on the X-axis. All sites plotted are significant at 5% FDR, and the horizontal dashed line represents the significance threshold for each bin. **(B)** Methylation levels across all samples for the gene *Lhx9.* The height of the bar represents percentage methylation from 0 to 100%. Gray represents missing data. **(C)** Average methylation levels in *C4a* at chr17:34960471. **(D)** Expression levels of *C4a* in RNA-seq dataset. **(E)** Expression levels of *C4a* measured by quantitative PCR. **(F)** Methylation levels of *Selenbp2* at chr3:94499361. **(G,H)** Expression levels of *Selenbp2* in BXD dataset **(G)** and by quantitative PCR **(H)**. Error bars represent the standard deviation. F, females; M, males.

To identify sex-specific methylation sites, we examined how DNA methylation patterns differed between females and males. As discussed above, we used a *t*-test to compare average methylation levels of reads from each group, but in this sex-specific comparison, we compared groups of females and males independent of genotype. We selected a cutoff of *P* < 0.001 and a difference in methylation between the sexes greater than 20% (FDR <5%), which showed a strong enrichment of sex-specific methylation sites in the X chromosome (56% of sites; Figure 3A). Using these criteria, we found 1,113 cytosines (0.026%) with sex-specific methylation, associated with a total of 396 genes. This corresponded to 1,082 CGs (0.14%), 7 CHGs and 24 CHHs. As an example, we found several cytosines differentially methylated in the gene encoding LIM homeobox protein 9 (*Lhx9*) on chromosome 1 (Figure 3B). For each site, the height of each bar represents the average methylation levels of 10 or more CpGs, between 0 and 100% methylation. Notably, a previous study found that knock-out mice for this gene show infertility in both sexes, impaired gonad formation, and female-like genitalia in genetically male mice [15,16]. We examined cytosines differentially methylated between the sexes using the Genomic Regions Enrichment of Annotations Tool (GREAT), and found an enrichment in genes associated with regulation of histone methylation (*P* = 8.32 × 10^-11^), regulation of bone mineralization (*P =* 1.16 × 10^-8^), abnormal genitalia (*P* = 3.6870 × 10^-16^), decreased aggression towards other mice (*P* = 8.33 × 10^-4^) and X-linked inheritance (*P* = 1.56 × 10^-128^). A complete list of the 396 genes showing sex-specific methylation is provided in Additional file 4.

To determine whether sex-specific methylation coincides with expression differences, we examined liver expression levels in two published datasets, a BXD mouse cross [16], and a BXD F1 RNA-seq dataset from our laboratory [17]. We compared expression levels between females and males of the genes with sex-specific methylation. In the BXD cross, 237 genes out of the 396 differentially methylated genes were represented in the array, and 97 of these genes (41% of all genes represented) were differentially expressed between females and males using a *t-*test (*P* < 0.05). Of these, 30 genes (13% of all genes) were differentially expressed over 1.2-fold at FDR <5%. In the RNA-seq dataset, 45 out of the 396 differentially methylated genes were represented in the data. Of these, 13 genes (29%) were differentially expressed between females and males over 1.2-fold (*P* < 0.05), and 5 of the genes (11%) were differentially expressed at FDR <5%.

We carried out additional validation experiments to examine expression levels of nine genes differentially methylated between females and males. We measured expression levels using quantitative PCR (qPCR) on liver cDNA from 10 female and 6 male F1 mice, and compared expression levels between the two groups using a two-tailed *t-*test. For example, *C4a* was differentially methylated, and we found that it was differentially expressed between females and males using both RNA-seq and qPCR (Figure 3C-E). Consistent with this finding, a previous study found that an open chromatin structure in males correlated with increased C4a protein levels in the liver [18]. Also consistent with previous data [19], the gene *Selenbp2*, which encodes the major hepatic target for acetaminophen, Selenium-binding protein 2, was differentially methylated, and we also found that it was differentially expressed between males and females in the published BXD dataset and in our qPCR validation (Figure 3F-H). Overall, four out of nine genes measured by qPCR were differentially expressed between the two groups (Figure S4 in Additional file 5). Although the remaining genes were not statistically significant at *P* < 0.05, they showed a trend towards differences between the two groups. It is possible that we did not have sufficient power to detect significant differences between the two groups using 16 F1 mice, as we did observe significant differences in the larger BXD expression array dataset. It is also possible that differences in methylation levels in some genes have functional consequences early in development but not in the adult animal, as suggested by large differences in methylation levels in *Lxh9*, but no significant differences in gene expression by qPCR in the adult liver. Thus, our findings suggest that DNA methylation is possibly a mechanism used to regulate sex-specific expression in a fraction of genes differentially methylated in the liver. A complete list of the genes differentially methylated and expressed is provided in Additional file 4.

### Imprinting

**Figure 4:**
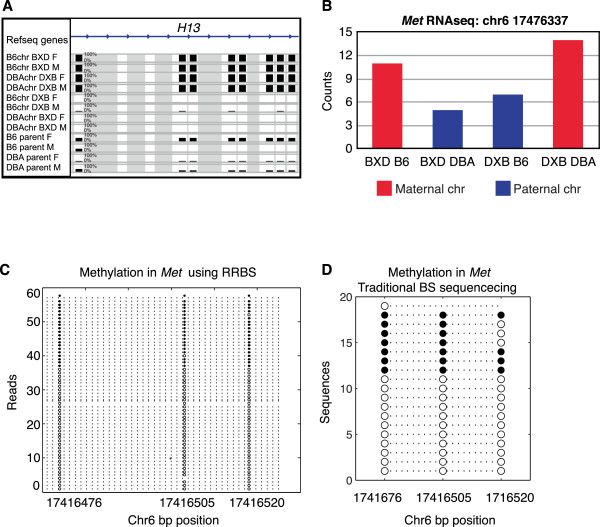
**Imprinting. (A)** Methylation levels across all samples, showing imprinting of the gene *H13.* The height of the bar represents percentage methylation from 0 to 100%. Gray represents missing data. **(B)** Expression levels of *Met* measured by RNA-seq. **(C)** Methylation levels for an allelically methylated region (AMR) in the gene *Met* measured by RRBS sequencing. Each line represents CpGs in a sequencing read; open circles are unmethylated and filled circles are methylated CpGs. Reads are shown on the Y-axis and the genomic location of CpGs is shown on the X-axis. **(D)** Traditional bisulfite sequencing (BS) in the *Met* AMR*.* Sequences in different bacterial clones are on the Y-axis and the genomic location of CpGs is on the X-axis.

DNA methylation is one of the mechanisms involved in genomic imprinting, whereby one of the two copies of a gene is expressed or silenced depending on the parent of origin. To identify imprinted genes, we first used the presence of polymorphic SNPs between the parental strains B6 and DBA in the sequencing reads to determine whether individual reads were derived from the father or the mother for each of the F1 mice. We then compared the average methylation level of maternal and paternal reads using a *t-*test and selected sites at the 5% FDR cutoff. Using these criteria, we identified three previously known imprinted genes, *H13*, *Impact* and *Snrpn*, but no novel imprinted genes. As an example, methylation patterns in the *H13* gene (Figure 4A) show that maternal chromosomes are highly methylated (B6 chromosomes in BXD mice and DBA chromosomes in DXB mice, shown in rows 1 to 4) while paternal chromosomes have low methylation levels (DBA chromosomes in BXD mice and B6 chromosomes in the DXB mice, shown in rows 5 to 8). The height of each bar represents the average methylation levels of 10 or more CpGs, between 0 and 100% methylation.

One likely reason that we were unable to find additional imprinted genes using this approach is that polymorphic reads represent only approximately 3 to 7% of the sequencing data we collected. To overcome this limitation, we used the method described by Fang *et al*. [20] to identify allelically methylated regions (AMRs), which is not limited to the use of polymorphic reads. The AMR approach uses a likelihood test to identify intervals of the genome that appear to have two alleles, each with a distinct methylation pattern. We selected AMRs where one allele was highly methylated and the other was not, where the difference in methylation between the two alleles was at least 50%, and where the AMR was defined by at least 10 consecutive CpGs. Since the AMR approach is independent of genotype, it can identify both (a) allele-specific methylation that is dependent on the genotype of the DNA strand, and (b) imprinted regions that are dependent on the parental origin and independent of genotype. To focus on imprinted regions, we selected AMRs that were present in both the F1 mice and the parental mice, since the parental mice are inbred and both alleles have the same genotype on both alleles, and hence AMRs could not be caused by allele-specific methylation. Using this approach, we identified 123 putative imprinted protein-coding and microRNA genes, which included 11 known imprinted genes (enrichment *P* = 7.02 × 10^-11^). This list provides a comprehensive set of putative imprinted loci, many of which have not been previously reported (Additional file 6).

To determine if these sites are associated with parent-of-origin differential transcription, we tested for imprinting in the proximal genes using polymorphic reads from RNA-seq data in the F1 mice. Of the 123 genes, 32 were represented with 10 counts or more in the RNA-seq data. We tested for imprinting using Fisher’s exact test and found 10 (31%) of these had significantly different levels between the paternal and maternal copies (*P* < 0.05). As an example, we identified *Met* as a putative imprinted gene because there was an AMR in seven of the eight total parental and F1 samples (Figure 4C), and we confirmed *Met* imprinting using RNA-seq (Figure 4B). Furthermore, we carried out independent validation of our RRBS results using traditional bisulfite sequencing of the five putative novel imprinted genes *Met, Nsd1, Vps37b, Oprd1* and *Mapk15*. We found that *Met* was differentially methylated using traditional bisulfite sequencing (Figure 4D), closely resembling the methylation patterns we observed in the RRBS data (Figure 4C). Similarly, the putative novel imprinted genes *Nsd1, Vps37b, Oprd1* and *Mapk15* were differentially methylated using traditional bisulfite sequencing (Additional file 7), consistent with our RRBS results.

A complete list of the known and novel imprinted genes we identified can be found in Additional file 6. Notably, we did not find imprinted genes or AMRs that were specific to either females or males. It is possible that we did not find evidence for imprinting in additional genes, or sex-specific differences due to lack of power. For instance, the known imprinted gene *Copg2* was part of the list of 32 genes represented with 10 or more counts, but it did not pass the significance threshold. Furthermore, it is also possible that we did not find additional imprinted genes because of tissue-specific differences, such that additional imprinted genes may not necessarily be imprinted in the liver.

### Intergenerational DNA methylation

Intergenerational epigenetic marks are epigenetic marks, such as DNA methylation, that can be inherited from one organism to another. To examine the conservation and level of variation in intergenerational DNA methylation, we used a binomial test to compare methylation levels in B6 parents with those in B6 chromosomes in their F1 offspring, and in DBA parents with the DBA chromosomes in their F1 offspring. We found a total of 2,068 (0.4%) intergenerational epimutations present in at least one parent-child pair that differed in methylation level by at least 50%, and were below the 5% confidence interval of the binomial test. On average, we found 223 epimutations between parent-child pairs. When divided by category, we found, on average, 133 epimutations in female parent-child pairs and 303 in males, 173 in B6 and 232 in DBA chromosomes, with no significant differences between the groups (Figure S6A in Additional file 8). To determine if these epimutations were reproducible or stochastic, we selected sites that were reproducible in at least one or two parent-F1 comparisons (Figure S6C,D in Additional file 8). We found 973 (0.2%) epimutations reproducible in at least one parent-F1 comparison, and 75 (0.02%) epimutations in at least two parent-F1 comparisons, excluding sites associated with known imprinted genes. Notably, we found reproducible epimutations in at least two parent-F1 pairs only in B6 chromosomes and not in DBA chromosomes, which may reflect the fact that we have higher power to detect B6 epimutations as the reference genome we are using was derived from a B6 mouse.

The genomic distribution of epimutations can be found in Figure S6C,D in Additional file 8. Epimutations reproducible in at least one parent-F1 pair were generally distributed throughout the genome, although they tended to cluster in specific regions, or 'hotspots'. Epimutations reproducible in at least two parent-F1 pairs mostly clustered in two 'epimutation hotspots'. The first was located in the potassium channel-encoding gene *Kcnip3* in chromosome 2, a gene involved in neuronal excitability that was previously associated with Alzheimer’s disease [21]. The second 'hotspot' was located the Obscurin gene (*Obscn*) in chromosome 11, a gene that may be involved in the formation of myofibrils. An example of a reproducible epimutation in the gene *Wdr63* is shown in Figure S6B in Additional file 8). We conclude that, overall, reproducible intergenerational epigenetic changes in DNA methylation are rare, and that DNA methylation marks tend to be highly conserved between parents and offspring.

## Discussion

In this study, we measured the extent of epigenetic variation among genetically distinct mice across two generations. We also surveyed expression data from similar samples to measure the degree to which these methylation differences are associated with expression changes. We found that DNA methylation was highly variable among genetically distinct individuals and differential methylation was strongly associated with polymorphic sites. We found thousands of cytosines differentially methylated between the parental mouse strains B6 and DBA (Figure 1B; Figure S2A in Additional file 2), and there was a strong enrichment for sites that are proximal to polymorphic alleles (Figure S2E,F in Additional file 2). Similarly, Xie *et al*. [4] found that 9.7% of CGs show allele-specific methylation between the more distantly related mouse strain 129X1/SvJ and the mouse subspecies CAST/EiJ. This higher rate supports the notion that increased genetic variation is strongly associated with increased epigenetic variation [22].

Beyond the measurement of the association of genetic and epigenetic changes, our study design also allowed us to observe differences in DNA methylation between identical chromosomes across generations. Strikingly, we found that intergenerational epigenetic changes were exceedingly rare. With the exception of sites that were affected by imprinting, only 75 others were found to have reproducible differences between parents and F1 hybrid offspring in at least two parent- F1 comparisons. Thus, our overall results support the notion that (a) DNA methylation is highly heritable in mice, (b) there are minimal *trans* effects in F1 hybrids compared to their inbred parents, and (c) the majority of reproducible DNA methylation differences that accumulate between genetically identical mice are due to parent-of-origin effects. This result contrasts with the occurrence rate in plants, where intergenerational epigenetic changes appear to be more common, and are associated with RNA interference pathways [14].

Previous studies that examined sex-specific DNA methylation differences have typically focused on the X chromosome and have failed to identify significant differences in autosomes. Here, we compared DNA methylation patterns in females and males and found sex-specific methylation differences in 1,113 cytosines (0.026%). We found that most of these (56%) were on the X chromosome, but we also observed a large proportion of sex-specific methylation sites in autosomes. For example, we identified multiple sex-specific methylation changes in the autosomal gene *Lhx9*, and mouse knock-outs of this gene display defects in fertility and the development of genitalia and gonads. We also found differential methylation and expression of *Maged1*, and mouse knock-outs for this gene display impaired sexual behavior, decreased social interactions and hyperphagia [23]. A subset of the 396 differentially methylated genes also showed sex-specific expression differences in a previously published BXD mouse intercross and RNA-seq data from BXD/DXB F1 mice (Additional file 4). In summary, our results suggest that sex-specific epigenetic differences are found throughout the genome and can influence gene expression and physiological phenotypes. Since knock-outs for differentially methylated genes such as *Lhx9* and *Maged1* are impaired in sexual development and behavior, it is possible that specific epigenetic changes involved in sexual dimorphism are established early in development and reinforced by DNA methylation differences in adult animals.

Aberrant inheritance and expression of imprinted genes can result in clinical phenotypes such as Prader-Willi, Angelman and Turner syndromes. Currently, there are 150 known imprinted genes in mice, and we sought to identify novel imprinted genes. Differentially methylated regions (DMRs) controlling imprinted genes may be established early in development (gametic or primary DMRs) and are maintained in adult tissues, or established after fertilization (secondary or somatic DMRs) and may be tissue-specific [24]. Here we used a new approach to identify AMRs in regions where the genotype of the two chromosomes is not necessarily different [20]. The method relies on the identification of regions where the methylation of reads segregate into two populations, one highly methylated and one unmethylated. Using this approach we were able to identify a total of 123 imprinted genes. These included 11 known imprinted genes and 112 novel protein coding and microRNA genes, and we validated 10 putative novel imprinted genes using RNA-seq. Future studies are needed to examine the phenotypic consequences of aberrant imprinting in these novel regions.

## Conclusion

Using RRBS sequencing in eight parental and F1 mice, we found novel imprinted genes, sex-specific DNA methylation in genes across the genome and allele-specific methylation. Our results strongly support the notion that, in mammals, the majority of variation in DNA methylation across individuals is associated with genetic differences, and is highly heritable from one generation to the next. Epigenetic factors have typically not been considered in genome-wide studies for complex traits, but our results suggest that DNA methylation is variable between individuals and is highly heritable, and hence can be a contributing factor to the heritability of traits. In the future it will be important to determine the contribution of epigenetic variation to the heritability of clinical traits, and the phenotypic impact of epigenetic variation on both molecular and complex clinical phenotypes.

## Materials and methods

### Mice

Mice from strains C57BL/6J and DBA/2J were obtained from the Jackson Laboratories (Bar Harbor, ME, USA) and bred to generate F1 mice using reciprocal crosses, then housed in pathogen-free conditions and according to NIH guidelines. We collected livers from mice at 16 weeks of age, froze them in liquid nitrogen and stored them at -80°C. The mice were fasted overnight for 16 hours prior to euthanasia.

### Accession numbers

RRBS sequencing data from this study, as well as all the results from strain-specific, allele-specific and sex-specific differentially methylated cytosines have been deposited in the NCBI GEO [25] under accession number [GEO:GSE53714].

### Reduced representation bisulfite sequencing libraries

We used a previously described method for constructing RRBS libraries [26]. In brief, we isolated genomic DNA from livers using a phenol-chloroform extraction, digested genomic DNA using MspI restriction enzyme (NEB, Ipswich, MA, USA), then carried out end-repair/adenylation (NEB) and ligation with pre-methylated adapters (Illumina, San Diego, CA, USA). We selected fragments 250 to 350 bp in size using 2% agarose gel electrophoresis, purified the DNA from the gel fragments (QIAGEN, Valencia, CA, USA) and carried out bisulfite treatment on the DNA (Milipore, Billerica, MA, USA) followed by PCR amplification.

### Alignment

We aligned reads using BS Seeker [12] to the mm9 mouse reference genome, keeping uniquely aligned reads.

### SNP calling

We pooled data from B6, DBA and BXD F1 samples for A, C, G and T counts at each position observed. We called SNPs for loci with a minimum of six counts and a minor allele frequency of 10%, excluding C/T SNPs, and cross-checked our calls with public sequencing data for these strains [13]. We observed a total of 24,132 SNPs in our data. For each sample, approximately 3 to 7% of the reads were polymorphic.

### Simulations

We simulated methylation counts to estimate FDR in methylation levels. For a specific site, we simulated binomially distributed data using the total number of counts (*n*) and the average methylation level (*p*) at that site, using *binornd* with parameters *n* and *p* in MATLAB. We took the ratio between the number of sites that were significant by chance in the simulation data (*x*) over the number of significant sites in the real data (*y*) as the simulation-based FDR (*x*/*y*).

### Binomial test

We selected data from cytosines covered by 10 or more reads. To test for differences in methylation at specific sites between individual samples, we used the binomial distribution with parameters *p* = percent methylation at that site, and *n* = total number of counts, and estimated 95% confidence intervals using *binofit* in MATLAB [14]. Sites were considered differentially methylated between two samples if the mean methylation of each sample was outside of the 95% confidence interval of the other sample, and if the difference between the mean methylation *delta* was >50%. This corresponded to a simulation-based FDR <1%.

### Inter-strain versus intra-strain variance

To compare the distribution of the variance in methylation levels, we first computed the variance in methylation levels for each cytosine across RRBS libraries of difference strains (inter-strain variance), the variance in methylation levels between samples derived from different mice of the same strain (intra-strain variance for biological replicates), or the variance in methylation levels between different libraries made from the same mouse genomic DNA sample (technical replicates). We then compared the distribution of these variances using a KS test (*kstest2* function in MATLAB), and visualized the distributions using the cumulative distribution function (*cdfplot* function in MATLAB). We calculated the mean of the variance in methylation across all cytosines to compare the fold difference between the distributions, and determined that the mean of the inter-strain variance was nine-fold higher than the intra-strain variance in biological replicates. We also selected data only from CG cytosines to compute the mean of the variance in methylation levels, and found that the mean variance in CG methylation was 2.4-fold higher between strains than between biological replicates.

### Allele-specific methylation

We tested for allele-specific methylation between groups of B6 and DBA samples using a *t*-test. We used our called SNPs to determine if reads were derived from B6 or DBA chromosomes, and determined the percentage methylation levels for each. We compared percentage methylation levels between B6 and DBA chromosomes using a *t-*test. Sites were considered differentially methylated if *P* < 0.05 and *delta* between B6 and DBA groups was >50%. This corresponded to FDR <0.05% based on 1,000 simulations.

### Sex differences

We tested for sex differences using a *t-*test as described above, but we tested for differences in sites using reads derived from females versus males. Sites were considered differentially methylated if *P* < 0.001 and *delta* between females and males was >20%, which corresponded to FDR <5%. We used the mm9 genome annotation [27] to identify 396 genes located within 10 kb of differentially methylated sites.

### Quantitative PCR (qPCR)

We purified RNA from livers of 10 female and 6 male F1 mice using Trizol (Life Technologies, Grand Island, NY, USA) and synthesized cDNA using 1 μg of RNA and a cDNA synthesis kit (Life Technologies), then diluted the cDNA 20 times and used 3 μl of the diluted cDNA per qPCR reaction. For each gene target, we measured each sample in triplicate using the KAPA Biosystems SYBR Green Mix (Wilmington, MA, USA) in a 12 μl reaction volume and the following conditions: 95°C for 5 minutes, and 50 cycles of 95°C for 15 seconds, 60°C for 15 seconds, and 72°C for 15 seconds. We determined the absolute quantity of each transcript using a standard curve in the Roche Light Cycler 480 program, and normalized the quantity of each sample relative to the quantity of the house-keeping gene *Rpl*. We compared average levels of each transcript between females and males using a two-tailed *t-*test.

### Imprinting

We used our called SNPs to determine whether reads in F1 mice were derived from B6 or DBA chromosomes, and classified reads as derived from maternal or paternal for both reciprocal F1 types BXD and DXB. We compared percentage methylation levels at individual sites in maternal versus paternal chromosomes using a *t-*test as described above. Sites were considered imprinted if *P* < 1 × 10^-6^, which corresponded to FDR <50% based on 1,000 simulations, but resulted in only three known imprinted genes.

### Allelically methylated regions

AMRs were identified by the computational method described by Fang *et al*. [20] with the tool 'amrfinder'. The tool uses a fixed-width window (that is, fixed number of CpG sites) to identify AMRs genome-wide. For each window, the likelihood values of observed reads from bisulfite sequencing are calculated by assuming the region is or is not an AMR. Then the likelihood ratio test is employed to determine the more appropriate model for the region. The underlying probabilistic model describes the degree to which reads appear to have two distinct methylation patterns and is independent of genotype. We selected AMRs called using a window size of 10 consecutive CpGs, *P* < 0.01 and *delta* between the two methylation patterns was greater than 50%, and considered the AMR as a putative imprinted region if it was present in at least 6 of the 8 total samples, including parents and F1 mice. Enrichment in imprinted genes was calculated using the hypergeometric test, *p =* 1-hygecdf in MATLAB.

### RNA-seq

#### Imprinting

RNA-seq data from the livers of 11 mice were recently published by Lagarrigue *et al*. [17], and included 6 BXD, 5 DXB, 6 females and 5 males. The authors used SNPs in polymorphic reads to determine parental origin of the read, and we used these RNA-seq counts to look for evidence for imprinting using a Fisher’s exact test, where the variables are cross direction (BXD, DXB) and genotype (B6, DBA), and selected specific sites where FDR <5% using the false discovery approach introduced by Storey [28], and where the fold difference between the two sexes was greater than 1.2-fold.

#### Sex differences

To test for sex differences in the RNA-seq dataset, we used a *t-*test to compare counts at specific sites between female and male F1 mice, and estimated FDR using the Storey method [28].

### Expression in BXD intercross

We obtained Agilent array expression data from a published BXD intercross composed of a total of 122 mice, parental, F1 and F2 mice, 72 females and 50 males [16]. We used a *t-*test to compare females versus males for individual genes, and estimated FDR using the Storey method [28]. We selected genes at a FDR <5% cutoff, and where the fold difference between females and males was greater than 1.2-fold.

### GREAT

We looked for enriched biological processes and phenotype annotations using GREAT [29]*.* To annotate fragments with sex-specific methylation, we used the list of DNA fragments that were differentially methylated between females and males as our test list, and all the fragments represented in our RRBS data as the reference list.

### Traditional bisulfite sequencing

We carried out bisulfite treatment using 1 μg of liver genomic DNA from BXD or DXB F1 male mice. We designed degenerate primers (IDT) to amplify bisulfite-treated DNA with the Bisulfite Primer Seeker program from Zymo (Irvine, CA, USA). We amplified each target gene with MyTaq (Bioline), using 2 μL of DNA in 50 PCR reaction volume, and the following PCR conditions: 95°C for 2 min; 2 cycles of 95°C for 30s, 62°C for 90s and 62°C for 2 min; 2 cycles of 95°C for 30s, 61°C for 90s and 61°C for 2 min; 2 cycles of 95°C for 30s, 60°C for 90s and 60°C for 2 min; 2 cycles of 95°C for 30s, 59°C for 90s and 60°C for 2 min; 31 cycles of 95°C for 30s, 58°C for 90s and 60°C for 2 min; final extension at 60°C for 15 min. We cloned PCR products from each sample using the TOPO cloning kit for sequencing (Life Technologies, Grand Island NY, USA) according to the manufacturer’s instructions. We plated transformed bacterial on Luria broth plates containing 50 μg/ml kanamycin and 20 μg/ml X-gal overnight at 37°C in the dark. We picked 15-20 white colonies from each transformation and submitted the clones for Sanger sequencing to Genewiz (La Jolla, CA, USA). We aligned the sequences using multiple alignment in the CLC Main Workbench program.

## Abbreviations

AMR: allelically methylated region; B6: C57BL/6J mouse strain; BXD: C57BL/6J x DBA/2J F1 mouse, where C57BL/6J was the female parent and DBA/2J was the male parent; DBA: DBA/2J mouse strain; DMR: differentially methylated region; DXB: DBA/2J x C57BL/6J F1 mouse, where DBA/2J was the female parent and C57BL/6J was the male parent; FDR: false discovery rate; KS: Kolmogorov-Smirnov; PCR: polymerase chain reaction; qPCR: quantitative PCR; RRBS: reduced representation bisulfite sequencing; SNP: single nucleotide polymorphism.

## Competing interests

The authors declare that they have no competing interests.

## Authors’ contributions

LDO performed the analyses and wrote the paper. LR constructed the sequencing libraries. LM bred the mice and collected the tissues. FF and ADS performed the analyses on the AMRs. FH aligned the RNA-seq data. LDO and NC performed traditional bisulfite sequencing experiments. AJL, MP and LDO conceived the project, and helped craft and edit the manuscript. MP directed the project. All authors read and approved the final manuscript.

## Supplementary Material

Additional file 1: Figure S1 RRBS data quality. **(A)** Total number of reads and uniquely aligned reads in each RRBS library. **(B)** Mappability of each library, or fraction of uniquely aligned reads by BS-Seeker. **(C)** Average coverage in cytosines. **(D)** Sequencing error rate in reads as the percentage of reads that do not match expected genotypes in libraries from B6 and DBA mice (Reads with error). **(E,F)** Genome-wide average methylation levels for each sample in **(E)** CG, **(F)** CHG and CHH contexts. Click here for file

Additional file 2: Figure S2 Strain specific differences. **(A)** Differentially methylated cytosines in B6 and DBA male parents. The number of cytosines differentially methylated in each 100-kb bin is shown on the Y-axis and the genomic position of the bin is on the X-axis. All sites plotted are significant at 1% FDR, and the horizontal dashed line represents the significance threshold for each bin. **(B,C)** Fraction of differentially methylated cytosines in each context in **(B)** female and **(C)** male B6 and DBA strains. **(D)** Overlap of differentially methylated cytosines identified in B6 and DBA female mice, or B6 and DBA male mice. **(E,F)** The distribution of the number of differentially methylated cytosines in **(E)** B6 and DBA females and **(F)** B6 and DBA males. The cumulative distribution function for the number of cytosines differentially methylated is shown for non-polymorphic bins, and polymorphic bins containing at least one SNP. Click here for file

Additional file 3: Figure S3 Cytosine methylation clusters by genotype of chromosomes. Hierarchical clustering of samples based on methylation levels from 38,427 cytosine sites. Individual B6 or DBA chromosomes in BXD mice were determined based on the genotype of polymorphic SNPs present in the read. Click here for file

Additional file 4: Table S1 Genes differentially methylated by sex. Click here for file

Additional file 5: Figure S4 Validation of sexual dimorphism by qPCR. **(A-I)** Expression levels measured by qPCR on mouse liver cDNA for nine genes differentially methylated between females and males. Expression levels of each gene are plotted on the Y-axis relative to the house-keeping gene *Rpl*. Each bar represents the average of 10 female (F) and 6 male (M) mice. Click here for file

Additional file 6: Table S2 Imprinted genes identified through AMRs. Click here for file

Additional file 7: Figure S5 Validation of AMRs by traditional bisulfite sequencing. **(A-D)** Traditional bisulfite sequencing for AMRs in **(A) ***Nsd1*, **(B) ***Vps37b*, **(C) ***Oprd1* and **(D) ***Mapk15.* Sequences in different bacterial clones are on the Y-axis and the genomic location of CpGs is on the X-axis. Open circles are unmethylated and filled circles are methylated CpGs. Click here for file

Additional file 8: Figure S6 Intergenerational methylation differences. **(A)** Average number of epimutations between parent and F1s. The average of parent-F1 comparisons is shown for all, or specifically for comparisons between females, males, B6 or DBA chromosomes. **(B)** Methylation levels across all samples in the gene *Wdr63*, showing variation in B6 chromosomes from different samples. BXD are F1 mice of B6 female and DBA male parents, DXB are F1 mice of DBA female of B6 male parents. The height of the bar represents percentage methylation from 0 to 100%. Gray represents missing data. **(C,D)** Reproducible epimutations across the genome identified in at least one **(C)** or two **(D)** parent- F1 comparisons. The number of epimutations in each 100-kb bin is shown on the Y-axis and the genomic position of the bin is on the X-axis. The horizontal dashed line represents the significance threshold for each bin. Click here for file
