# Supplementary figures and images for: Intergenerational genomic DNA methylation patterns in mouse hybrid strains
Source: Genome Biol. 2014 Apr 30;15(5):R68. doi: 10.1186/gb-2014-15-5-r68 (PMC4076608; doi:10.1186/gb-2014-15-5-r68)

**Figure S1. RRBS data quality**

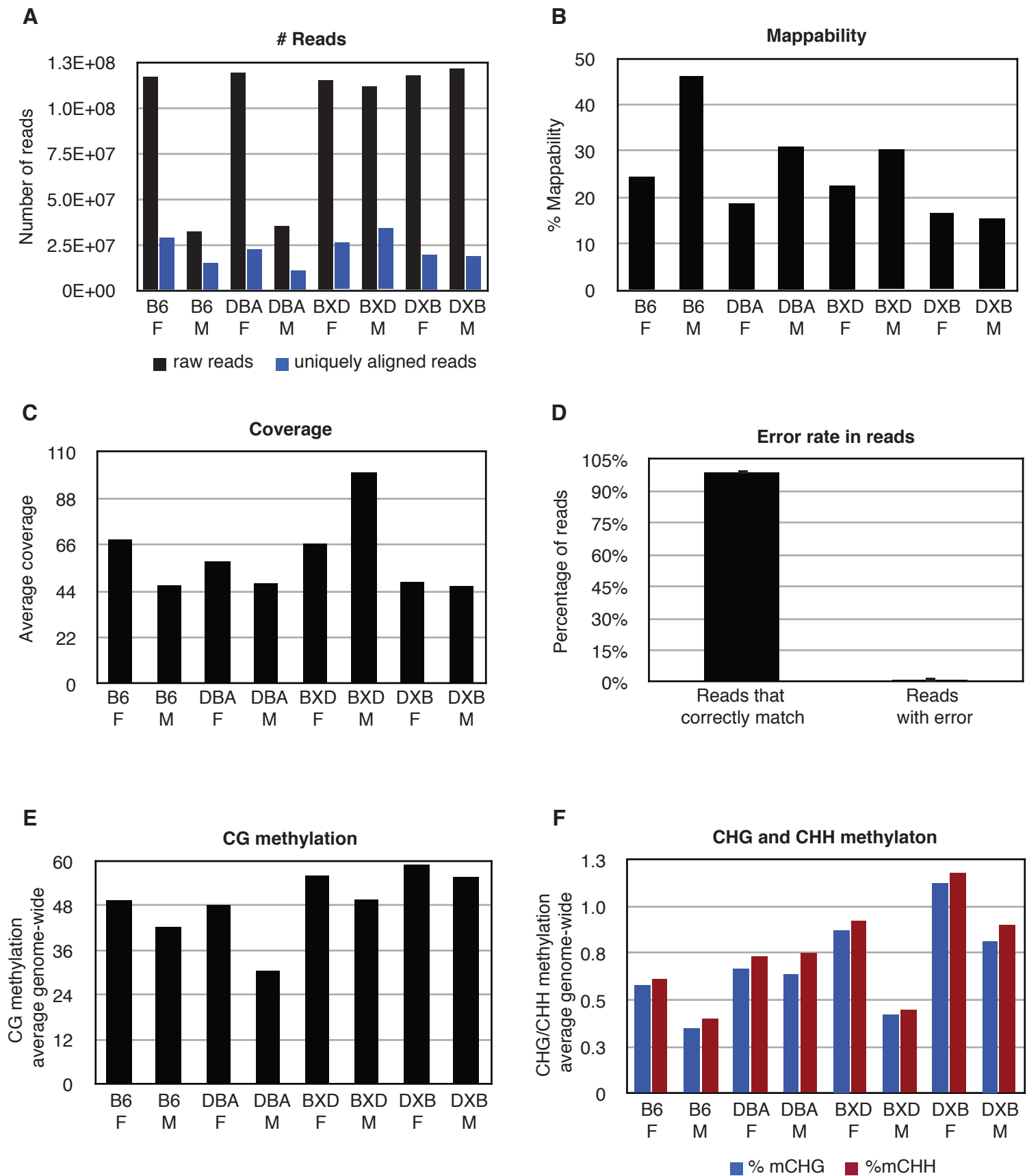

Supplement: Additional file 1: Figure S1 — RRBS data quality. (A) Total number of reads and uniquely aligned reads in each RRBS library. (B) Mappability of each library, or fraction of uniquely aligned reads by BS-Seeker. (C) Average coverage in cytosines. (D) Sequencing error rate in reads as the percentage of reads that do not match expected genotypes in libraries from B6 and DBA mice (Reads with error). (E,F) Genome-wide average methylation levels for each sample in (E) CG, (F) CHG and CHH contexts. [file gb-2014-15-5-r68-S1.pdf]

**Figure S2. Strain-specific differences**

**A**

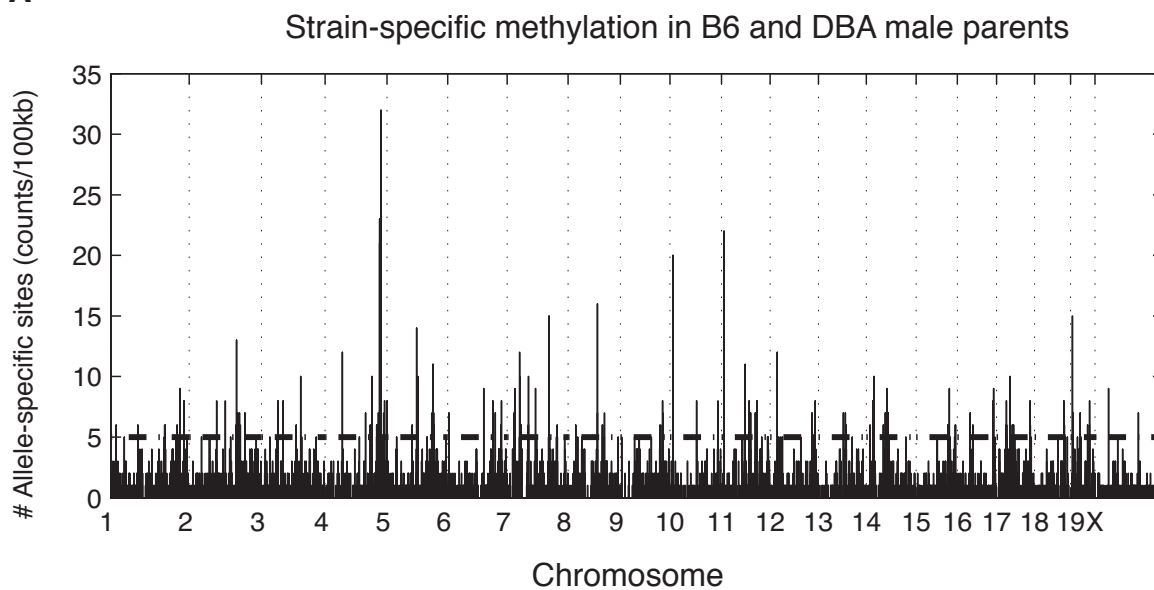

**B**

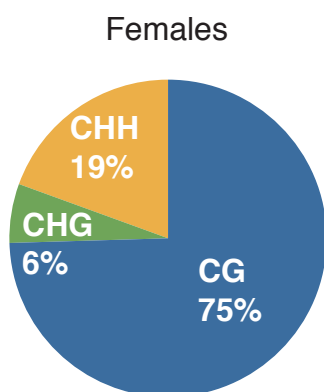

**C**

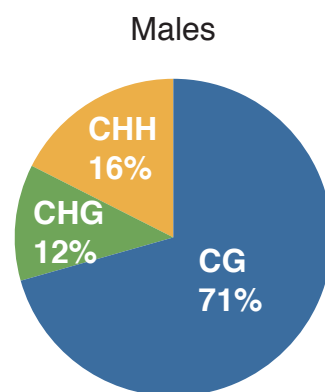

**D**

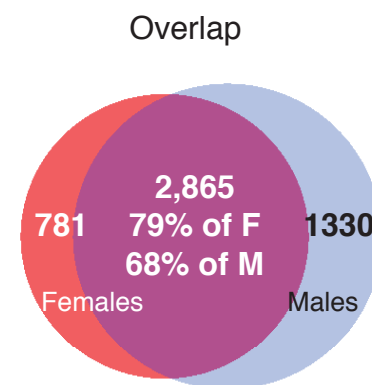

**E**

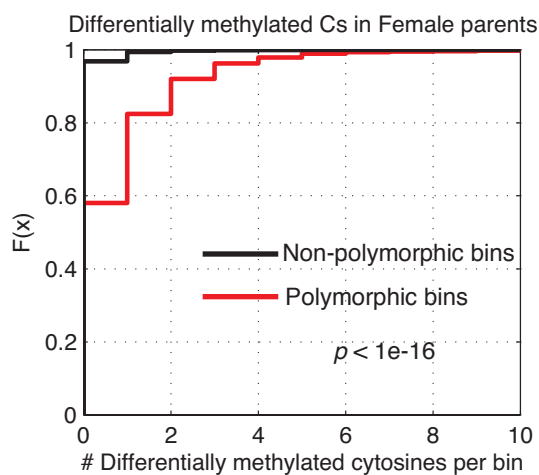

**F**

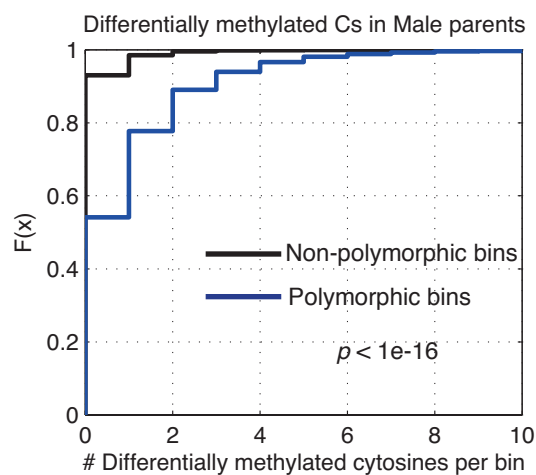

Supplement: Additional file 2: Figure S2 — Strain specific differences. (A) Differentially methylated cytosines in B6 and DBA male parents. The number of cytosines differentially methylated in each 100-kb bin is shown on the Y-axis and the genomic position of the bin is on the X-axis. All sites plotted are significant at 1% FDR, and the horizontal dashed line represents the significance threshold for each bin. (B,C) Fraction of differentially methylated cytosines in each context in (B) female and (C) male B6 and DBA strains. (D) Overlap of differentially methylated cytosines identified in B6 and DBA female mice, or B6 and DBA male mice. (E,F) The distribution of the number of differentially methylated cytosines in (E) B6 and DBA females and (F) B6 and DBA males. The cumulative distribution function for the number of cytosines differentially methylated is shown for non-polymorphic bins, and polymorphic bins containing at least one SNP. [file gb-2014-15-5-r68-S2.pdf]

**Figure S3. Cytosine methylation clusters by genotype of chromosome**

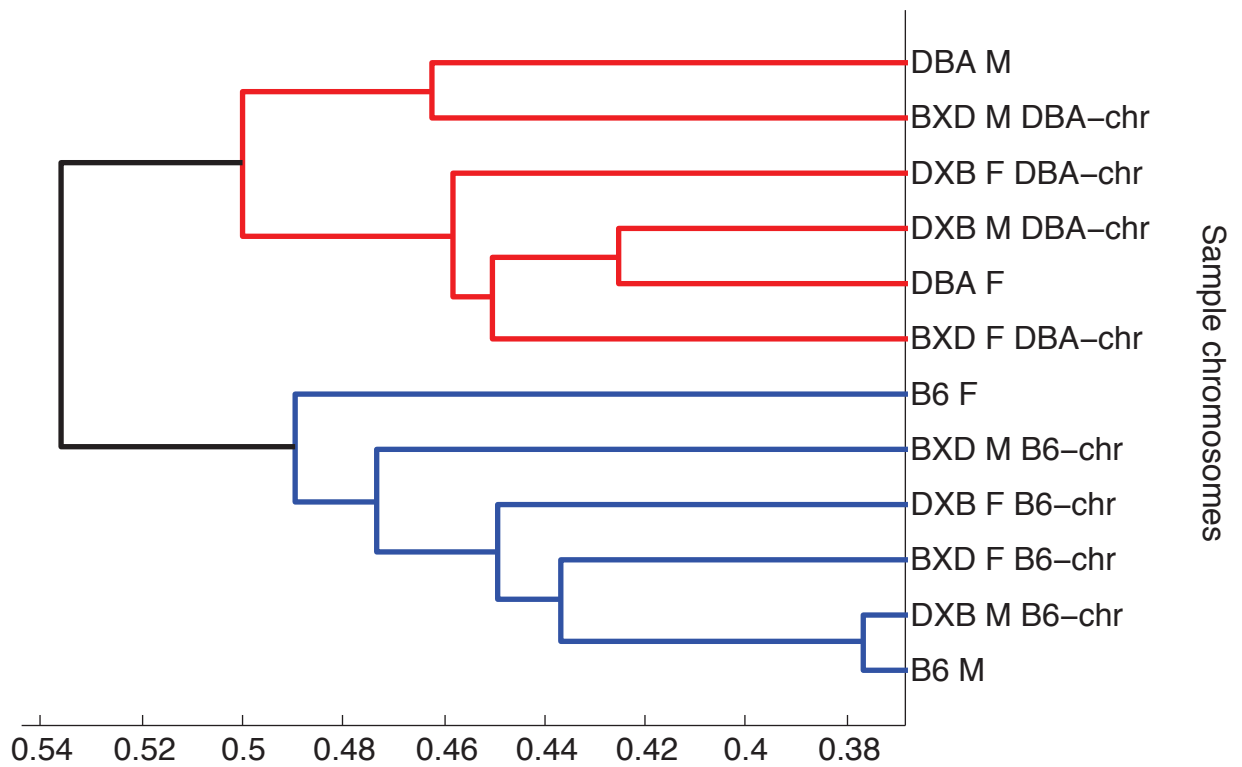

Supplement: Additional file 3: Figure S3 — Cytosine methylation clusters by genotype of chromosomes. Hierarchical clustering of samples based on methylation levels from 38,427 cytosine sites. Individual B6 or DBA chromosomes in BXD mice were determined based on the genotype of polymorphic SNPs present in the read. [file gb-2014-15-5-r68-S3.pdf]

**Figure S4. Validation of sexual dimorphism by qPCR**

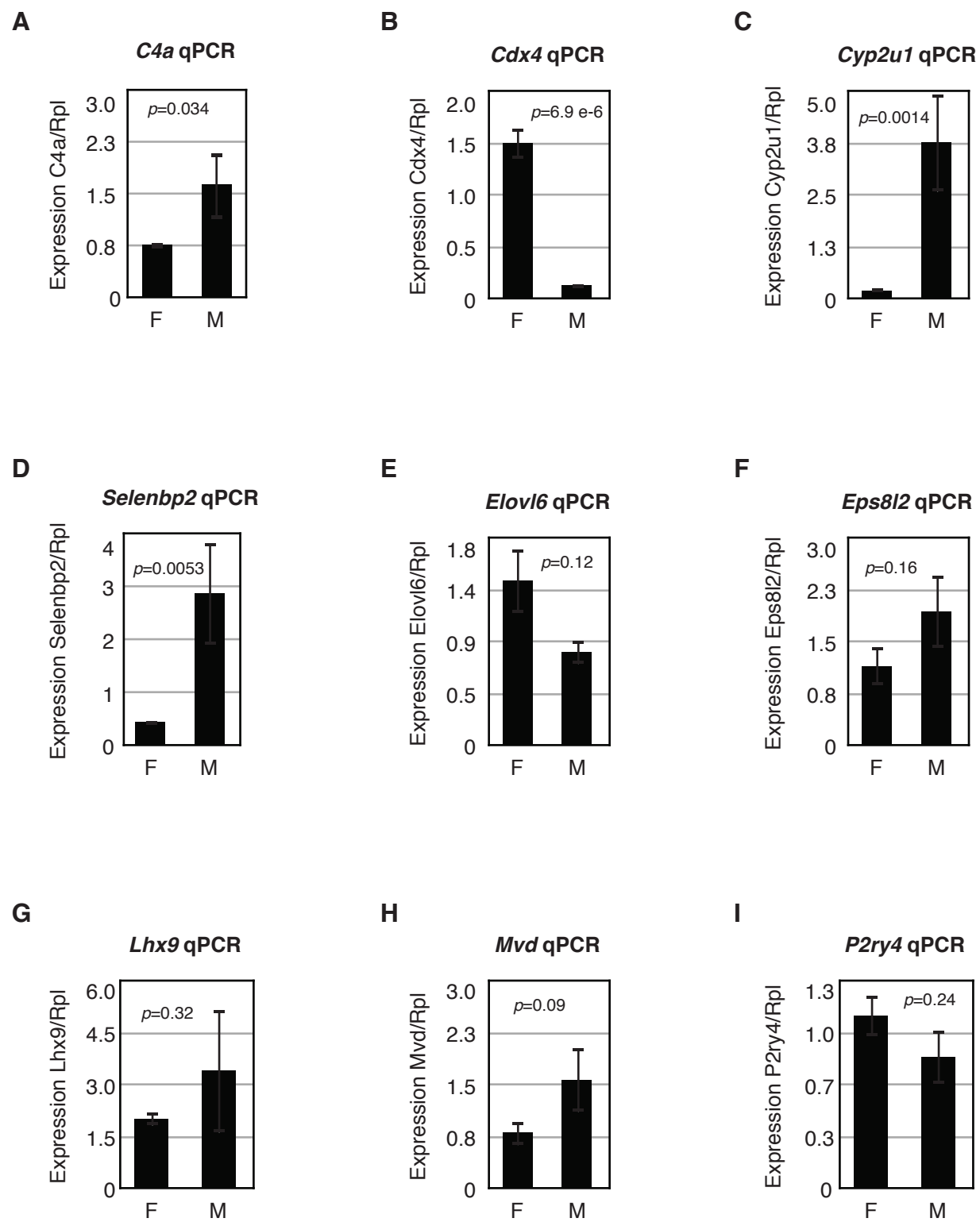

Supplement: Additional file 5: Figure S4 — Validation of sexual dimorphism by qPCR. (A-I) Expression levels measured by qPCR on mouse liver cDNA for nine genes differentially methylated between females and males. Expression levels of each gene are plotted on the Y-axis relative to the house-keeping gene Rpl. Each bar represents the average of 10 female (F) and 6 male (M) mice. [file gb-2014-15-5-r68-S5.pdf]

Figure S5. Validation of AMRs by traditional bisulfite sequencing

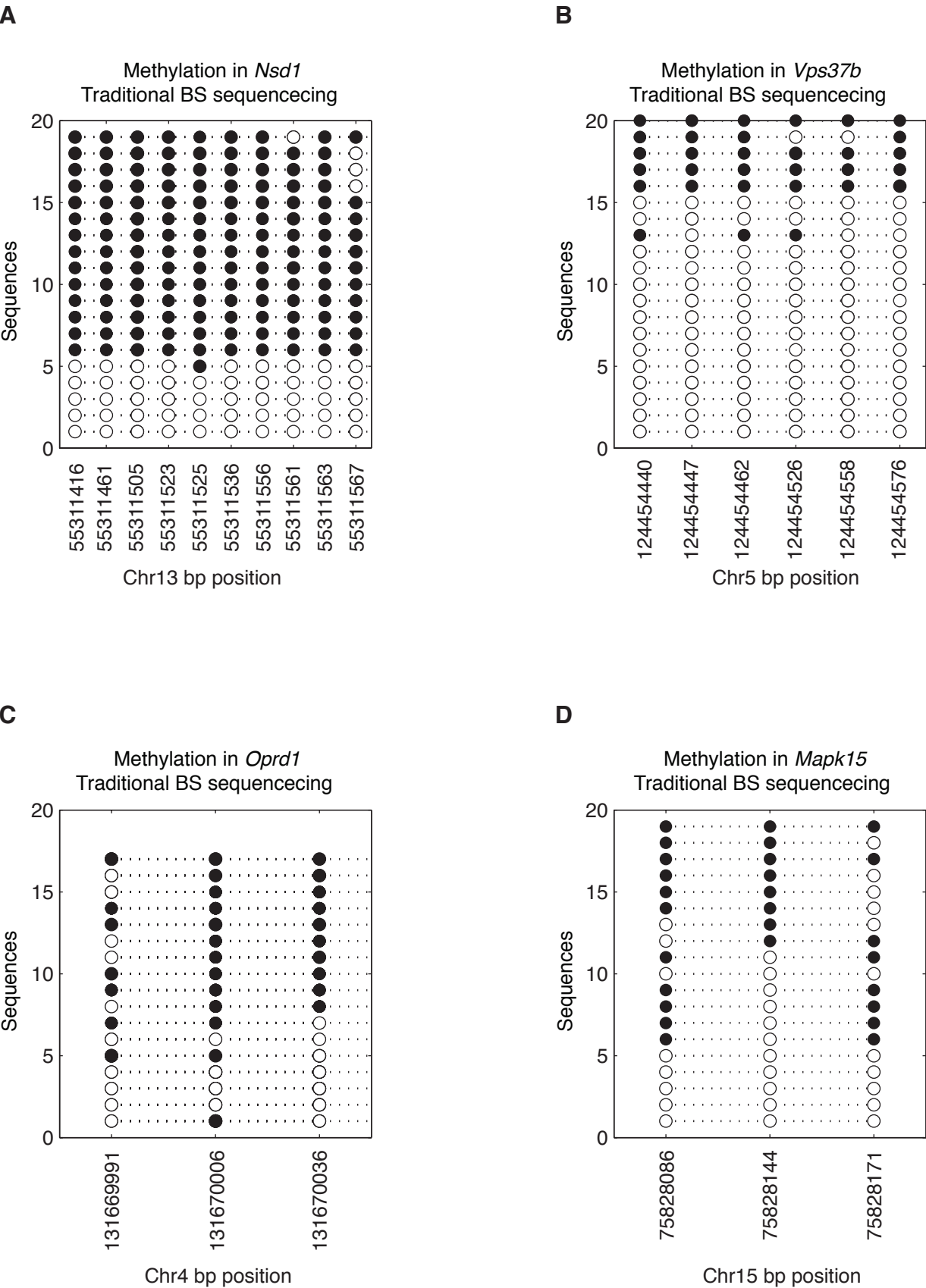

Supplement: Additional file 7: Figure S5 — Validation of AMRs by traditional bisulfite sequencing. (A-D) Traditional bisulfite sequencing for AMRs in (A) Nsd1, (B) Vps37b, (C) Oprd1 and (D) Mapk15. Sequences in different bacterial clones are on the Y-axis and the genomic location of CpGs is on the X-axis. Open circles are unmethylated and filled circles are methylated CpGs. [file gb-2014-15-5-r68-S7.pdf]

Figure S6. Intergenerational methylation differences

A

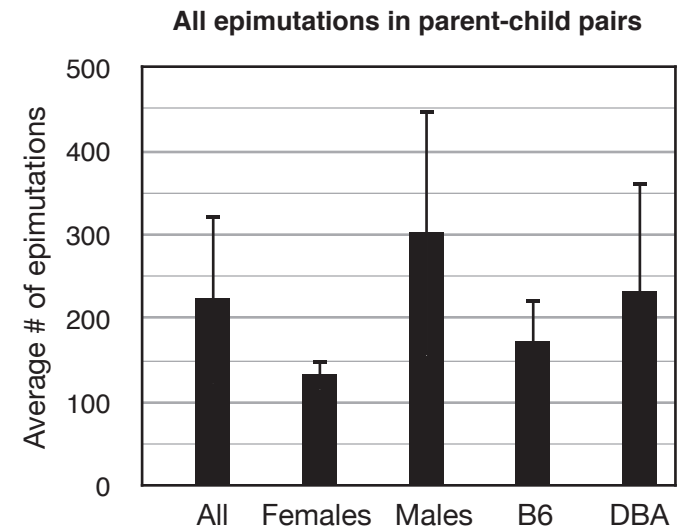

B

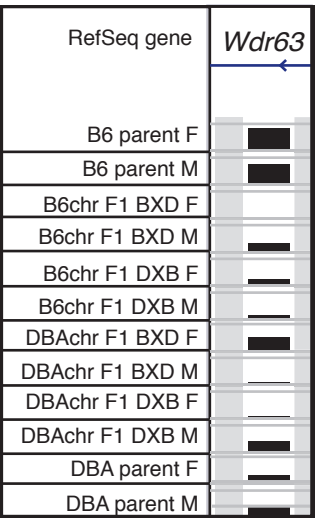

C

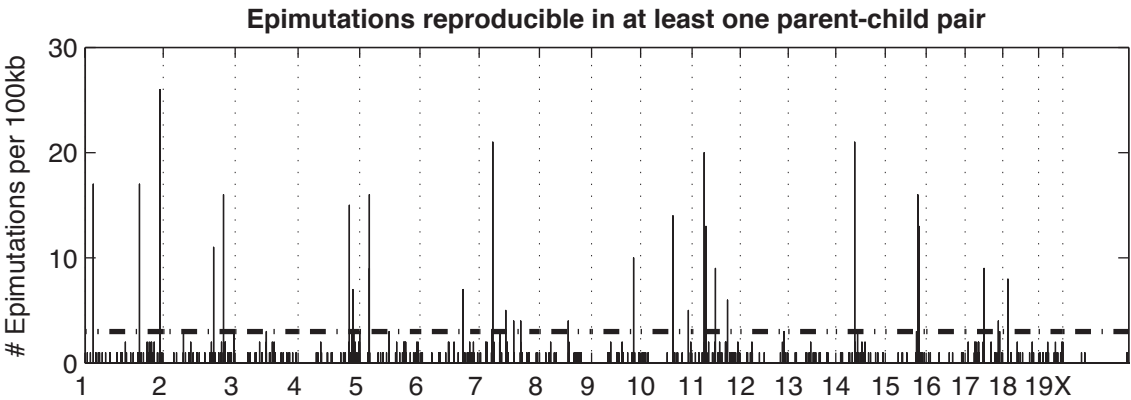

D

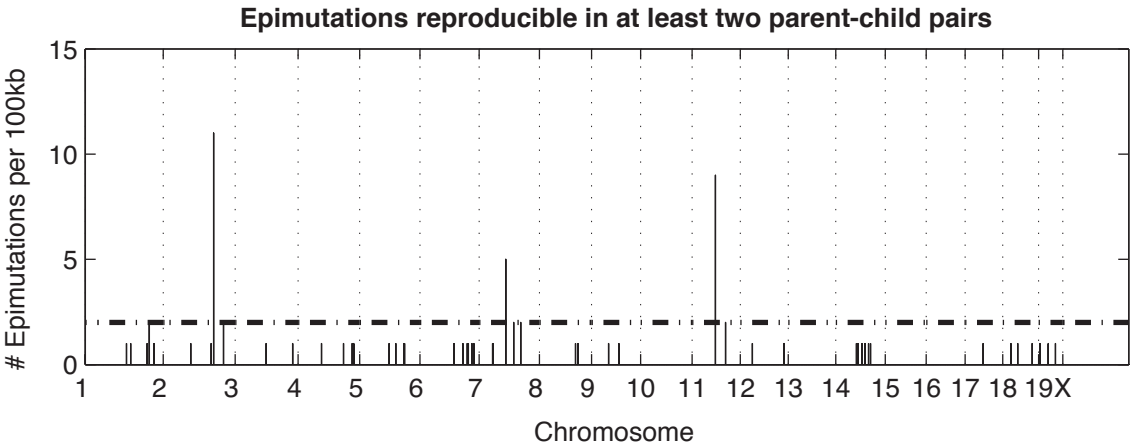

Supplement: Additional file 8: Figure S6 — Intergenerational methylation differences. (A) Average number of epimutations between parent and F1s. The average of parent-F1 comparisons is shown for all, or specifically for comparisons between females, males, B6 or DBA chromosomes. (B) Methylation levels across all samples in the gene Wdr63, showing variation in B6 chromosomes from different samples. BXD are F1 mice of B6 female and DBA male parents, DXB are F1 mice of DBA female of B6 male parents. The height of the bar represents percentage methylation from 0 to 100%. Gray represents missing data. (C,D) Reproducible epimutations across the genome identified in at least one (C) or two (D) parent- F1 comparisons. The number of epimutations in each 100-kb bin is shown on the Y-axis and the genomic position of the bin is on the X-axis. The horizontal dashed line represents the significance threshold for each bin. [file gb-2014-15-5-r68-S8.pdf]
