# Supplementary material for: Intergenerational genomic DNA methylation patterns in mouse hybrid strains
Source: Genome Biol. 2014 Apr 30;15(5):R68. doi: 10.1186/gb-2014-15-5-r68 (PMC4076608; doi:10.1186/gb-2014-15-5-r68)
Supplement: Additional file 4: Table S1 — Genes differentially methylated by sex. [file gb-2014-15-5-r68-S4.doc]

| **Table S1. Genes differentially methylated by sex** | | | | | | | | | |
| --- | --- | --- | --- | --- | --- | --- | --- | --- | --- |
| Gene symbol | Chr | *p* for differential expression in BXD | Fold female/male in BXD | Direction of expression in BXD | %FDR in BXD | *p* for differential expression in RNA-seq | Fold female/male in RNA-seq | Direction of expression in RNAseq | %FDR in RNA-seq |
| *Etnk2* | 1 |  |  |  |  | 5.06E-03 | 2.54 |  | 10.59 |
| *Fzd7* | 1 |  |  |  |  |  |  |  |  |
| *Lhx9* | 1 |  |  |  |  |  |  |  |  |
| *Slc4a3* | 1 |  |  |  |  |  |  |  |  |
| *Sox13* | 1 | 1.71E-07 | 0.80 | Male is higher | 1.66E-04 | 1.61E-02 | 0.43 | Male is higher | 26.29 |
| *6820408C15Rik* | 2 |  |  |  |  |  |  |  |  |
| *A530013C23Rik* | 2 |  |  |  |  |  |  |  |  |
| *Cbfa2t2* | 2 |  |  |  |  |  |  |  |  |
| *Cebpb* | 2 |  |  |  |  |  |  |  |  |
| *Mir684-1* | 2 |  |  |  |  |  |  |  |  |
| *Phf20* | 2 |  |  |  |  |  |  |  |  |
| *Cyp2u1* | 3 | 1.10E-12 | 0.76 | Male is higher | 3.99E-09 | 3.66E-02 | 0.42 | Male is higher | 33.59 |
| *Elovl6* | 3 | 1.59E-05 | 1.52 | Female is higher | 0.01 |  |  |  |  |
| *Magi3* | 3 |  |  |  |  |  |  |  |  |
| *Mfn1* | 3 |  |  |  |  |  |  |  |  |
| *Selenbp2* | 3 | 2.07E-15 | 0.50 | Male is higher | 1.50E-11 |  |  |  |  |
| *1700125D06Rik* | 4 |  |  |  |  |  |  |  |  |
| *Bsdc1* | 4 |  |  |  |  |  |  |  |  |
| *Casp9* | 4 | 1.26E-02 | 0.90 | Male is higher | 2.55 | <1E-16 | 0.50 | Male is higher | <1E-16 |
| *Cela2a* | 4 |  |  |  |  |  |  |  |  |
| *Clspn* | 4 |  |  |  |  |  |  |  |  |
| *Ddost* | 4 |  |  |  |  | 2.19E-02 | 1.62 | Female is higher | 30.65 |
| *Gmeb1* | 4 |  |  |  |  |  |  |  |  |
| *Hcrtr1* | 4 |  |  |  |  |  |  |  |  |
| *Npr2* | 4 | 3.50E-02 | 1.10 | Female is higher | 5.25 | 1.97E-02 | 1.76 | Female is higher | 28.86 |
| *Pef1* | 4 |  |  |  |  | <1E-16 | 2.00 | Female is higher | <1E-16 |
| *Pink1* | 4 |  |  |  |  | 1.85E-02 | 2.00 | Female is higher | 28.49 |
| *S100pbp* | 4 |  |  |  |  |  |  |  |  |
| *Thrap3* | 4 |  |  |  |  |  |  |  |  |
| *Tssk3* | 4 |  |  |  |  |  |  |  |  |
| *Yars* | 4 | 4.17E-03 | 0.90 | Male is higher | 0.98 |  |  |  |  |
| *Cyth3* | 5 |  |  |  |  |  |  |  |  |
| *Mrpl33* | 5 | 1.73E-03 | 0.91 | Male is higher | 0.47 |  |  |  |  |
| *Rbks* | 5 |  |  |  |  |  |  |  |  |
| *Stim2* | 5 |  |  |  |  |  |  |  |  |
| *Trim56* | 5 | 3.34E-03 | 1.21 | Female is higher | 0.82 |  |  |  |  |
| *Creld1* | 6 | 3.28E-07 | 0.82 | Male is higher | 2.81E-04 |  |  |  |  |
| *Il17rc* | 6 | 3.16E-02 | 1.06 | Female is higher | 4.90 |  |  |  |  |
| *Prrt3* | 6 |  |  |  |  |  |  |  |  |
| *Ruvbl1* | 6 |  |  |  |  |  |  |  |  |
| *Sec61a1* | 6 |  |  |  |  |  |  |  |  |
| *Deaf1* | 7 |  |  |  |  |  |  |  |  |
| *Eps8l2* | 7 | 5.64E-09 | 0.65 | Male is higher | 6.85E-06 |  |  |  |  |
| *Pdilt* | 7 |  |  |  |  |  |  |  |  |
| *Prr12* | 7 |  |  |  |  |  |  |  |  |
| *Prrg2* | 7 |  |  |  |  |  |  |  |  |
| *Snrnp70* | 7 |  |  |  |  |  |  |  |  |
| *Taldo1* | 7 | 2.82E-02 | 0.93 | Male is higher | 4.51 |  |  |  |  |
| *Tmem80* | 7 |  |  |  |  |  |  |  |  |
| *9330133O14Rik* | 8 |  |  |  |  |  |  |  |  |
| *Agpat6* | 8 |  |  |  |  |  |  |  |  |
| *D030016E14Rik* | 8 | 3.09E-02 | 0.95 | Male is higher | 4.89 |  |  |  |  |
| *Dpep1* | 8 |  |  |  |  |  |  |  |  |
| *Gse1* | 8 |  |  |  |  |  |  |  |  |
| *Insr* | 8 |  |  |  |  |  |  |  |  |
| *Mlf1ip* | 8 |  |  |  |  |  |  |  |  |
| *Mvd* | 8 | 1.30E-03 | 1.36 | Female is higher | 0.41 |  |  |  |  |
| *Nfix* | 8 |  |  |  |  | 1.30E-02 | 2.26 | Female is higher | 22.38 |
| *Rwdd4a* | 8 |  |  |  |  |  |  |  |  |
| *Tacc1* | 8 |  |  |  |  |  |  |  |  |
| *Wdr59* | 8 |  |  |  |  |  |  |  |  |
| *1300017J02Rik* | 9 | 2.03E-02 | 0.88 | Male is higher | 3.61 |  |  |  |  |
| *1700102P08Rik* | 9 |  |  |  |  |  |  |  |  |
| *Arhgap42* | 9 |  |  |  |  |  |  |  |  |
| *Bbs9* | 9 |  |  |  |  |  |  |  |  |
| *Cldn18* | 9 | 1.23E-05 | 0.85 | Male is higher | 0.01 |  |  |  |  |
| *Hyou1* | 9 |  |  |  |  |  |  |  |  |
| *Pdcd7* | 9 |  |  |  |  |  |  |  |  |
| *Smad3* | 9 | 3.57E-05 | 0.86 | Male is higher | 0.02 |  |  |  |  |
| *Trf* | 9 |  |  |  |  |  |  |  |  |
| *Usp4* | 9 | 1.81E-02 | 1.07 | Female is higher | 3.37 |  |  |  |  |
| *Zbtb16* | 9 |  |  |  |  | 3.66E-03 | 6.75 | Female is higher | 9.77 |
| *4930404N11Rik* | 10 |  |  |  |  |  |  |  |  |
| *F630110N24Rik* | 10 |  |  |  |  |  |  |  |  |
| *Fzr1* | 10 |  |  |  |  |  |  |  |  |
| *Nab2* | 10 |  |  |  |  |  |  |  |  |
| *Pias4* | 10 |  |  |  |  |  |  |  |  |
| *Pwp2* | 10 |  |  |  |  |  |  |  |  |
| *Stat6* | 10 |  |  |  |  |  |  |  |  |
| *Trappc10* | 10 |  |  |  |  |  |  |  |  |
| *Zbtb7a* | 10 |  |  |  |  |  |  |  |  |
| *Baiap2* | 11 |  |  |  |  |  |  |  |  |
| *Caskin2* | 11 |  |  |  |  |  |  |  |  |
| *Cdc42ep4* | 11 |  |  |  |  |  |  |  |  |
| *Ddx42* | 11 | 8.15E-03 | 1.08 | Female is higher | 1.74 |  |  |  |  |
| *Ftsj3* | 11 | 6.97E-03 | 0.91 | Male is higher | 1.56 |  |  |  |  |
| *Helz* | 11 | 3.09E-03 | 1.15 | Female is higher | 0.78 |  |  |  |  |
| *Ksr1* | 11 |  |  |  |  |  |  |  |  |
| *Myh4* | 11 |  |  |  |  |  |  |  |  |
| *Nptx1* | 11 |  |  |  |  |  |  |  |  |
| *Psmc5* | 11 |  |  |  |  |  |  |  |  |
| *Pyy* | 11 |  |  |  |  |  |  |  |  |
| *Tsen54* | 11 |  |  |  |  |  |  |  |  |
| *6430527G18Rik* | 12 |  |  |  |  |  |  |  |  |
| *Chga* | 12 | 5.81E-09 | 0.74 | Male is higher | 6.51E-06 |  |  |  |  |
| *Itpk1* | 12 |  |  |  |  |  |  |  |  |
| *Mir153* | 12 |  |  |  |  |  |  |  |  |
| *Prima1* | 12 |  |  |  |  |  |  |  |  |
| *Etohd2* | 13 |  |  |  |  |  |  |  |  |
| *Faf2* | 13 |  |  |  |  |  |  |  |  |
| *Isca1* | 13 |  |  |  |  |  |  |  |  |
| *Mier3* | 13 |  |  |  |  |  |  |  |  |
| *Otp* | 13 |  |  |  |  |  |  |  |  |
| *Rnf44* | 13 | 2.77E-04 | 0.90 | Male is higher | 0.10 |  |  |  |  |
| *Slc12a7* | 13 |  |  |  |  |  |  |  |  |
| *Tbce* | 13 | 2.60E-03 | 0.89 | Male is higher | 0.66 |  |  |  |  |
| *Zcchc6* | 13 |  |  |  |  |  |  |  |  |
| *Zfp366* | 13 |  |  |  |  |  |  |  |  |
| *Ap1g2* | 14 |  |  |  |  |  |  |  |  |
| *Jph4* | 14 | 4.96E-09 | 0.87 | Male is higher | 6.56E-06 |  |  |  |  |
| *Lats2* | 14 |  |  |  |  |  |  |  |  |
| *Scara5* | 14 |  |  |  |  |  |  |  |  |
| *Tsc22d1* | 14 |  |  |  |  |  |  |  |  |
| *Zfp503* | 14 | 3.03E-04 | 1.25 | Female is higher | 0.11 |  |  |  |  |
| *Grhl2* | 15 |  |  |  |  |  |  |  |  |
| *Lalba* | 15 | 4.18E-02 | 1.19 |  | 5.97 |  |  |  |  |
| *Tspyl5* | 15 |  |  |  |  |  |  |  |  |
| *3110052M02Rik* | 17 |  |  |  |  |  |  |  |  |
| *C4a* | 17 |  |  |  |  | 1.09E-04 | 0.005 | Male is higher | 0.53 |
| *Cpne5* | 17 |  |  |  |  |  |  |  |  |
| *Dom3z* | 17 | 3.54E-10 | 0.83 | Male is higher | 6.45E-07 |  |  |  |  |
| *Skiv2l* | 17 |  |  |  |  |  |  |  |  |
| *Stk19* | 17 | 1.71E-11 | 0.77 | Male is higher | 4.98E-08 |  |  |  |  |
| *Tdrd6* | 17 |  |  |  |  |  |  |  |  |
| *Acaa2* | 18 | 1.85E-02 | 0.91 | Male is higher | 3.42 |  |  |  |  |
| *Mir1948* | 18 |  |  |  |  |  |  |  |  |
| *Myo5b* | 18 | 1.42E-04 | 0.78 | Male is higher | 0.06 | <1E-16 | 2.00 | Female is higher | <1E-16 |
| *Scarna17* | 18 |  |  |  |  |  |  |  |  |
| *2700081O15Rik* | 19 |  |  |  |  |  |  |  |  |
| *Fads2* | 19 | 1.28E-04 | 1.23 | Female is higher | 0.05 | <1E-16 | 0.50 | Male is higher | <1E-16 |
| *Frmd8* | 19 |  |  |  |  |  |  |  |  |
| *Gsto1* | 19 | 1.79E-02 | 1.19 | Female is higher | 3.39 |  |  |  |  |
| *Rtn3* | 19 |  |  |  |  |  |  |  |  |
| *Slc25a45* | 19 |  |  |  |  |  |  |  |  |
| *Tmem180* | 19 |  |  |  |  |  |  |  |  |
| *Vax1* | 19 |  |  |  |  |  |  |  |  |
| *1110012L19Rik* | X | 3.05E-11 | 1.25 | Female is higher | 7.40E-08 |  |  |  |  |
| *1700045I19Rik* | X | 8.72E-03 | 0.87 | Male is higher | 1.84 |  |  |  |  |
| *2010204K13Rik* | X |  |  |  |  |  |  |  |  |
| *2610018G03Rik* | X |  |  |  |  |  |  |  |  |
| *2610030H06Rik* | X |  |  |  |  |  |  |  |  |
| *2900002K06Rik* | X |  |  |  |  |  |  |  |  |
| *4930524L23Rik* | X |  |  |  |  |  |  |  |  |
| *4930567H17Rik* | X |  |  |  |  |  |  |  |  |
| *6720401G13Rik* | X | 2.45E-02 | 1.09 | Female is higher | 4.20 |  |  |  |  |
| *A230072E10Rik* | X |  |  |  |  |  |  |  |  |
| *Abcd1* | X |  |  |  |  |  |  |  |  |
| *Aff2* | X |  |  |  |  |  |  |  |  |
| *Aifm1* | X |  |  |  |  |  |  |  |  |
| *Alg13* | X |  |  |  |  |  |  |  |  |
| *Ankrd58* | X |  |  |  |  |  |  |  |  |
| *Ap1s2* | X |  |  |  |  |  |  |  |  |
| *Apoo* | X |  |  |  |  |  |  |  |  |
| *Ar* | X | 2.00E-06 | 1.19 | Female is higher | 1.39E-03 |  |  |  |  |
| *Araf* | X |  |  |  |  |  |  |  |  |
| *Arhgap36* | X |  |  |  |  |  |  |  |  |
| *Arhgap6* | X | 7.79E-06 | 1.32 | Female is higher | 4.37E-03 |  |  |  |  |
| *Arhgef6* | X |  |  |  |  |  |  |  |  |
| *Arhgef9* | X |  |  |  |  |  |  |  |  |
| *Armcx3* | X |  |  |  |  |  |  |  |  |
| *Armcx5* | X |  |  |  |  |  |  |  |  |
| *Armcx6* | X |  |  |  |  |  |  |  |  |
| *Arr3* | X |  |  |  |  |  |  |  |  |
| *Arx* | X |  |  |  |  |  |  |  |  |
| *Atg4a* | X |  |  |  |  |  |  |  |  |
| *Atp11c* | X | 6.66E-04 | 0.85 | Male is higher | 0.23 |  |  |  |  |
| *Atp6ap1* | X |  |  |  |  |  |  |  |  |
| *B630019K06Rik* | X |  |  |  |  |  |  |  |  |
| *Bcap31* | X |  |  |  |  |  |  |  |  |
| *Bcor* | X | 2.17E-02 | 1.08 | Female is higher | 3.76 |  |  |  |  |
| *Bcorl1* | X |  |  |  |  |  |  |  |  |
| *Brcc3* | X |  |  |  |  |  |  |  |  |
| *Brs3* | X | 1.73E-03 | 0.87 | Male is higher | 0.47 |  |  |  |  |
| *Brwd3* | X |  |  |  |  |  |  |  |  |
| *Btk* | X | 7.37E-03 | 1.09 | Female is higher | 1.60 |  |  |  |  |
| *C330007P06Rik* | X |  |  |  |  |  |  |  |  |
| *Cacna1f* | X | 7.69E-06 | 0.87 | Male is higher | 4.48E-03 |  |  |  |  |
| *Cask* | X | 1.36E-04 | 0.89 | Male is higher | 0.05 |  |  |  |  |
| *Ccdc22* | X |  |  |  |  |  |  |  |  |
| *Cdk16* | X |  |  |  |  |  |  |  |  |
| *Cdx4* | X | 1.59E-09 | 1.90 | Female is higher | 2.32E-06 |  |  |  |  |
| *Chst7* | X | 2.80E-05 | 1.29 | Female is higher | 0.01 |  |  |  |  |
| *Cited1* | X |  |  |  |  |  |  |  |  |
| *Cnksr2* | X |  |  |  |  |  |  |  |  |
| *Ctps2* | X | 2.70E-08 | 1.28 | Female is higher | 2.80E-05 |  |  |  |  |
| *Cxx1c* | X |  |  |  |  |  |  |  |  |
| *Dcaf12l1* | X |  |  |  |  |  |  |  |  |
| *Dcaf12l2* | X |  |  |  |  |  |  |  |  |
| *Ddx26b* | X |  |  |  |  |  |  |  |  |
| *Diap2* | X | 3.14E-04 | 1.17 | Female is higher | 0.11 |  |  |  |  |
| *Dlg3* | X |  |  |  |  |  |  |  |  |
| *Dnase1l1* | X | 6.95E-03 | 1.10 | Female is higher | 1.58 |  |  |  |  |
| *Dock11* | X | 5.39E-06 | 1.23 | Female is higher | 3.27E-03 |  |  |  |  |
| *Dusp9* | X |  |  |  |  |  |  |  |  |
| *E230019M04Rik* | X |  |  |  |  |  |  |  |  |
| *Ebp* | X | 4.03E-03 | 1.15 | Female is higher | 0.98 |  |  |  |  |
| *Eda* | X | 1.68E-03 | 1.10 | Female is higher | 0.47 |  |  |  |  |
| *Efnb1* | X |  |  |  |  |  |  |  |  |
| *Egfl6* | X |  |  |  |  |  |  |  |  |
| *Eif1ax* | X |  |  |  |  |  |  |  |  |
| *Elf4* | X |  |  |  |  |  |  |  |  |
| *Emd* | X |  |  |  |  |  |  |  |  |
| *Ercc6l* | X |  |  |  |  |  |  |  |  |
| *Esx1* | X |  |  |  |  |  |  |  |  |
| *F8a* | X |  |  |  |  |  |  |  |  |
| *Fam120c* | X |  |  |  |  |  |  |  |  |
| *Fam70a* | X |  |  |  |  |  |  |  |  |
| *Fancb* | X |  |  |  |  |  |  |  |  |
| *Fgf13* | X | 3.23E-02 | 1.19 | Female is higher | 4.89 |  |  |  |  |
| *Flna* | X | 1.44E-03 | 1.13 | Female is higher | 0.42 |  |  |  |  |
| *Fmr1* | X | 1.52E-05 | 1.16 | Female is higher | 0.01 |  |  |  |  |
| *Foxo4* | X |  |  |  |  |  |  |  |  |
| *Gdi1* | X |  |  |  |  |  |  |  |  |
| *Gdpd2* | X |  |  |  |  |  |  |  |  |
| *Gjb1* | X | 1.30E-02 | 1.14 | Female is higher | 2.59 |  |  |  |  |
| *Gla* | X |  |  |  |  |  |  |  |  |
| *Gm10474* | X |  |  |  |  |  |  |  |  |
| *Gm5643* | X |  |  |  |  |  |  |  |  |
| *Gm614* | X |  |  |  |  |  |  |  |  |
| *Gm6985* | X |  |  |  |  |  |  |  |  |
| *Gnl3l* | X |  |  |  |  |  |  |  |  |
| *Gpc3* | X |  |  |  |  |  |  |  |  |
| *Gpm6b* | X | 3.20E-02 | 1.31 | Female is higher | 4.90 |  |  |  |  |
| *Gpr50* | X |  |  |  |  |  |  |  |  |
| *Gpr64* | X |  |  |  |  |  |  |  |  |
| *Gprasp1* | X | 1.95E-03 | 1.23 | Female is higher | 0.52 |  |  |  |  |
| *Gprasp2* | X |  |  |  |  |  |  |  |  |
| *Gria3* | X |  |  |  |  |  |  |  |  |
| *Gspt2* | X |  |  |  |  |  |  |  |  |
| *Gyk* | X |  |  |  |  |  |  |  |  |
| *Hccs* | X |  |  |  |  |  |  |  |  |
| *Hdac6* | X |  |  |  |  |  |  |  |  |
| *Hdac8* | X |  |  |  |  |  |  |  |  |
| *Hnrnph2* | X |  |  |  |  |  |  |  |  |
| *Hprt* | X |  |  |  |  |  |  |  |  |
| *Hs6st2* | X |  |  |  |  |  |  |  |  |
| *Htatsf1* | X | 4.99E-04 | 1.19 | Female is higher | 0.17 |  |  |  |  |
| *Idh3g* | X |  |  |  |  |  |  |  |  |
| *Ids* | X |  |  |  |  |  |  |  |  |
| *Igbp1* | X |  |  |  |  |  |  |  |  |
| *Il13ra1* | X | 1.57E-06 | 1.29 | Female is higher | 0.00 |  |  |  |  |
| *Il2rg* | X |  |  |  |  |  |  |  |  |
| *Iqsec2* | X |  |  |  |  |  |  |  |  |
| *Irs4* | X | 2.29E-10 | 0.69 | Male is higher | 4.77E-07 |  |  |  |  |
| *Kcne1l* | X |  |  |  |  |  |  |  |  |
| *Kif4* | X | 1.58E-02 | 1.09 | Female is higher | 3.07 |  |  |  |  |
| *Kis2* | X |  |  |  |  |  |  |  |  |
| *Klhl4* | X |  |  |  |  |  |  |  |  |
| *Lamp2* | X |  |  |  |  |  |  |  |  |
| *Lancl3* | X |  |  |  |  |  |  |  |  |
| *Las1l* | X |  |  |  |  |  |  |  |  |
| *LOC100270707* | X |  |  |  |  |  |  |  |  |
| *Lonrf3* | X |  |  |  |  |  |  |  |  |
| *Maged1* | X | 1.02E-04 | 0.84 | Male is higher | 0.04 |  |  |  |  |
| *Magee1* | X | 1.53E-03 | 0.90 | Male is higher | 0.44 |  |  |  |  |
| *Mageh1* | X | 4.56E-03 | 1.13 | Female is higher | 1.05 |  |  |  |  |
| *Magix* | X |  |  |  |  |  |  |  |  |
| *Mecp2* | X |  |  |  |  |  |  |  |  |
| *Med12* | X |  |  |  |  |  |  |  |  |
| *Mid1ip1* | X | 3.93E-06 | 1.42 | Female is higher | 2.49E-03 |  |  |  |  |
| *Mir106a* | X |  |  |  |  |  |  |  |  |
| *Mir18b* | X |  |  |  |  |  |  |  |  |
| *Mir19b-2* | X |  |  |  |  |  |  |  |  |
| *Mir20b* | X |  |  |  |  |  |  |  |  |
| *Mir2137* | X |  |  |  |  |  |  |  |  |
| *Mir322* | X |  |  |  |  |  |  |  |  |
| *Mir351* | X |  |  |  |  |  |  |  |  |
| *Mir363* | X |  |  |  |  |  |  |  |  |
| *Mir450-1* | X |  |  |  |  |  |  |  |  |
| *Mir450-2* | X |  |  |  |  |  |  |  |  |
| *Mir450b* | X |  |  |  |  |  |  |  |  |
| *Mir503* | X |  |  |  |  |  |  |  |  |
| *Mir505* | X |  |  |  |  |  |  |  |  |
| *Mir542* | X |  |  |  |  |  |  |  |  |
| *Mir92-2* | X |  |  |  |  |  |  |  |  |
| *Morc4* | X |  |  |  |  |  |  |  |  |
| *Mospd2* | X | 1.47E-06 | 1.16 | Female is higher | 1.19E-03 |  |  |  |  |
| *Msl3* | X |  |  |  |  |  |  |  |  |
| *Msn* | X | 5.82E-15 | 0.61 | Male is higher | 2.82E-11 |  |  |  |  |
| *Mtap7d2* | X |  |  |  |  |  |  |  |  |
| *Mtcp1* | X | 4.66E-02 | 0.95 | Male is higher | 6.52 |  |  |  |  |
| *Mycs* | X |  |  |  |  |  |  |  |  |
| *Naa10* | X |  |  |  |  |  |  |  |  |
| *Ncrna00086* | X |  |  |  |  |  |  |  |  |
| *Ndufa1* | X | 1.03E-02 | 0.92 | Male is higher | 2.11 |  |  |  |  |
| *Ndufb11* | X |  |  |  |  |  |  |  |  |
| *Ngfrap1* | X | 1.37E-03 | 0.90 | Male is higher | 0.43 |  |  |  |  |
| *Nhsl2* | X |  |  |  |  |  |  |  |  |
| *Nlgn3* | X | 4.10E-02 | 1.09 | Female is higher | 6.03 |  |  |  |  |
| *Nono* | X | 2.98E-07 | 1.14 | Female is higher | 2.72E-04 |  |  |  |  |
| *Nrk* | X |  |  |  |  |  |  |  |  |
| *Nup62cl* | X |  |  |  |  |  |  |  |  |
| *Nxt2* | X |  |  |  |  |  |  |  |  |
| *Nyx* | X |  |  |  |  |  |  |  |  |
| *Ogt* | X | 9.75E-04 | 1.21 | Female is higher | 0.32 |  |  |  |  |
| *Ophn1* | X | 1.66E-06 | 1.17 | Female is higher | 0.00 |  |  |  |  |
| *Otud5* | X |  |  |  |  |  |  |  |  |
| *P2ry4* | X | 5.45E-24 | 0.56 | Male is higher | 7.93E-20 |  |  |  |  |
| *Pcdh19* | X |  |  |  |  |  |  |  |  |
| *Pdha1* | X | 7.38E-04 | 0.90 | Male is higher | 0.24 |  |  |  |  |
| *Pdk3* | X | 1.53E-02 | 1.10 | Female is higher | 3.01 |  |  |  |  |
| *Pdzd11* | X |  |  |  |  |  |  |  |  |
| *Pdzd4* | X |  |  |  |  |  |  |  |  |
| *Pgrmc1* | X | 9.37E-03 | 0.88 | Male is higher | 1.95 |  |  |  |  |
| *Phf6* | X |  |  |  |  |  |  |  |  |
| *Phf8* | X | 3.13E-02 | 0.92 | Male is higher | 4.90 |  |  |  |  |
| *Phka1* | X | 2.48E-02 | 0.95 | Male is higher | 4.21 |  |  |  |  |
| *Phka2* | X | 2.18E-03 | 1.13 | Female is higher | 0.57 |  |  |  |  |
| *Piga* | X |  |  |  |  |  |  |  |  |
| *Pim2* | X | 1.68E-02 | 0.94 | Male is higher | 3.23 |  |  |  |  |
| *Pin4* | X | 4.17E-02 | 0.95 | Male is higher | 6.01 |  |  |  |  |
| *Plp2* | X |  |  |  |  |  |  |  |  |
| *Pnma3* | X |  |  |  |  |  |  |  |  |
| *Pola1* | X | 1.50E-09 | 1.29 | Female is higher | 2.42E-06 |  |  |  |  |
| *Porcn* | X | 2.30E-04 | 0.90 | Male is higher | 0.09 |  |  |  |  |
| *Pou3f4* | X |  |  |  |  |  |  |  |  |
| *Ppp1r3f* | X |  |  |  |  |  |  |  |  |
| *Pqbp1* | X | 9.82E-05 | 0.86 | Male is higher | 0.04 |  |  |  |  |
| *Prkx* | X |  |  |  |  |  |  |  |  |
| *Prps2* | X |  |  |  |  |  |  |  |  |
| *Psmd10* | X |  |  |  |  |  |  |  |  |
| *Ptchd1* | X |  |  |  |  |  |  |  |  |
| *Rab33a* | X |  |  |  |  |  |  |  |  |
| *Rap2c* | X |  |  |  |  |  |  |  |  |
| *Rbbp7* | X |  |  |  |  |  |  |  |  |
| *Rbm10* | X |  |  |  |  |  |  |  |  |
| *Rbm3* | X |  |  |  |  |  |  |  |  |
| *Rbm41* | X |  |  |  |  |  |  |  |  |
| *Rbmx* | X | 4.10E-03 | 1.11 | Female is higher | 0.98 |  |  |  |  |
| *Renbp* | X |  |  |  |  |  |  |  |  |
| *Reps2* | X |  |  |  |  |  |  |  |  |
| *Ribc1* | X |  |  |  |  |  |  |  |  |
| *Rnf113a1* | X |  |  |  |  |  |  |  |  |
| *Rpgr* | X |  |  |  |  |  |  |  |  |
| *Rpl10* | X |  |  |  |  |  |  |  |  |
| *Rpl39* | X |  |  |  |  |  |  |  |  |
| *Rps4x* | X |  |  |  |  |  |  |  |  |
| *Rps6ka3* | X | 1.92E-02 | 0.93 | Male is higher | 3.50 |  |  |  |  |
| *Sat1* | X |  |  |  |  |  |  |  |  |
| *Sept6* | X |  |  |  |  |  |  |  |  |
| *Sh3kbp1* | X |  |  |  |  |  |  |  |  |
| *Shroom4* | X |  |  |  |  |  |  |  |  |
| *Siah1b* | X | 4.34E-02 | 1.08 | Female is higher | 6.14 |  |  |  |  |
| *Slc10a3* | X |  |  |  |  |  |  |  |  |
| *Slc25a5* | X |  |  |  |  |  |  |  |  |
| *Slc35a2* | X |  |  |  |  |  |  |  |  |
| *Slc38a5* | X |  |  |  |  |  |  |  |  |
| *Slc7a3* | X |  |  |  |  |  |  |  |  |
| *Slc9a6* | X | 7.17E-03 | 0.91 | Male is higher | 1.58 |  |  |  |  |
| *Smc1a* | X |  |  |  |  |  |  |  |  |
| *Sms* | X |  |  |  |  |  |  |  |  |
| *Snora69* | X |  |  |  |  |  |  |  |  |
| *Snord61* | X |  |  |  |  |  |  |  |  |
| *Snx12* | X | 3.15E-06 | 1.21 | Female is higher | 2.08E-03 |  |  |  |  |
| *Sox3* | X |  |  |  |  |  |  |  |  |
| *Spin2* | X |  |  |  |  |  |  |  |  |
| *Spin4* | X |  |  |  |  |  |  |  |  |
| *Srpk3* | X |  |  |  |  |  |  |  |  |
| *Ssr4* | X | 1.44E-03 | 0.90 | Male is higher | 0.43 |  |  |  |  |
| *Suv39h1* | X |  |  |  |  |  |  |  |  |
| *Syp* | X |  |  |  |  |  |  |  |  |
| *Taz* | X |  |  |  |  |  |  |  |  |
| *Tbc1d25* | X |  |  |  |  |  |  |  |  |
| *Tbc1d8b* | X |  |  |  |  |  |  |  |  |
| *Tceal1* | X |  |  |  |  |  |  |  |  |
| *Tceanc* | X |  |  |  |  |  |  |  |  |
| *Tcfe3* | X |  |  |  |  |  |  |  |  |
| *Thoc2* | X |  |  |  |  |  |  |  |  |
| *Timm17b* | X |  |  |  |  |  |  |  |  |
| *Timm8a1* | X |  |  |  |  |  |  |  |  |
| *Tmem164* | X |  |  |  |  |  |  |  |  |
| *Tmem28* | X |  |  |  |  |  |  |  |  |
| *Tmem35* | X |  |  |  |  |  |  |  |  |
| *Tmem47* | X |  |  |  |  |  |  |  |  |
| *Tsc22d3* | X |  |  |  |  |  |  |  |  |
| *Tspan7* | X |  |  |  |  |  |  |  |  |
| *Uba1* | X |  |  |  |  |  |  |  |  |
| *Ube2a* | X |  |  |  |  |  |  |  |  |
| *Ubl4* | X |  |  |  |  |  |  |  |  |
| *Ubqln2* | X |  |  |  |  |  |  |  |  |
| *Upf3b* | X |  |  |  |  |  |  |  |  |
| *Uprt* | X |  |  |  |  |  |  |  |  |
| *Usp11* | X |  |  |  |  |  |  |  |  |
| *Usp27x* | X |  |  |  |  |  |  |  |  |
| *Utp14a* | X |  |  |  |  |  |  |  |  |
| *Was* | X |  |  |  |  |  |  |  |  |
| *Wdr13* | X |  |  |  |  |  |  |  |  |
| *Xlr4b* | X |  |  |  |  |  |  |  |  |
| *Xlr4c* | X |  |  |  |  |  |  |  |  |
| *Zbtb33* | X | 2.67E-02 | 0.93 | Male is higher | 4.41 |  |  |  |  |
| *Zfp185* | X | 1.43E-03 | 1.12 | Female is higher | 0.43 |  |  |  |  |
| *Zfp275* | X | 1.31E-04 | 1.10 | Female is higher | 0.05 |  |  |  |  |
| *Zfp711* | X |  |  |  |  |  |  |  |  |
| *Zic3* | X |  |  |  |  |  |  |  |  |
| *Zmym3* | X |  |  |  |  |  |  |  |  |
| *Zrsr2* | X |  |  |  |  |  |  |  |  |
