# Supplementary material for: Intergenerational genomic DNA methylation patterns in mouse hybrid strains
Source: Genome Biol. 2014 Apr 30;15(5):R68. doi: 10.1186/gb-2014-15-5-r68 (PMC4076608; doi:10.1186/gb-2014-15-5-r68)
Supplement: Additional file 6: Table S2 — Imprinted genes identified through AMRs. [file gb-2014-15-5-r68-S6.doc]

| **Table S2. Imprinted genes identified through AMRs** | |
| --- | --- |
| **Gene symbol** | **Chr** |
| *1700048O20Rik* | 9 |
| *1810012P15Rik* | 11 |
| *2310021P13Rik* | 14 |
| *5930403L14Rik* | 4 |
| *6230400D17Rik* | 14 |
| *Acsf2* | 11 |
| *Actn1* | 12 |
| ***Airn*** | 17 |
| *AK004252* | 16 |
| *AK018074* | 14 |
| *AK018948* | 5 |
| *AK027935* | 2 |
| *AK039760* | 16 |
| *AK047558* | 4 |
| *AK048257* | 5 |
| *AK050283* | 13 |
| *AK053386* | 13 |
| *AK083203* | 4 |
| *AK085136* | 2 |
| *AK145379* | 7 |
| *AK158482* | 18 |
| *AK196141* | 11 |
| *AK204442* | 14 |
| *Arl9* | 5 |
| *Asp3* | 4 |
| *Asprv1* | 6 |
| *Atp5h* | 11 |
| *B4galnt1* * | 10 |
| *Bace1* | 9 |
| *Bcl7c* | 7 |
| ***Begain*** | 12 |
| *C130050O18Rik* | 5 |
| *C530008M17Rik* | 5 |
| *Ccdc158* | 5 |
| *Cela1* | 15 |
| *Chad* | 11 |
| *Chchd1* | 10 |
| ***Copg2*** | 6 |
| *Cpsf3l* * | 4 |
| *Ctdsp1* | 1 |
| *Ctf1* | 7 |
| *Ctf2* | 7 |
| *Cuedc1* | 11 |
| *Cyp26c1* | 19 |
| *Dapk2* | 9 |
| *Ddit3* | 10 |
| *DQ716672* | 17 |
| *Dvl1* * | 4 |
| *Dysf* | 6 |
| *Ednrb* | 14 |
| *Fam132b* | 1 |
| *Fam83h* | 15 |
| *Fut11* | 14 |
| *Gabbr2* | 4 |
| *Gltpd1* * | 4 |
| *Gm11961* | 11 |
| ***Gnas*** | 2 |
| *Gpr179* | 11 |
| *Gpr97* | 8 |
| ***H19*** | 7 |
| *Il12a* | 3 |
| *Ilkap* | 1 |
| ***Impact*** | 18 |
| *Insc* | 7 |
| *Kctd2* * | 11 |
| *Kiaa0913* | 14 |
| *Klb* | 5 |
| *Lpp* | 16 |
| *Lrp12* | 15 |
| *Mapk15* | 15 |
| *Mars* * | 10 |
| ***Mest*** | 6 |
| *Met* * | 6 |
| *Mir1966* | 8 |
| *Mir26b* | 1 |
| ***Mir335*** | 6 |
| *Mir762* | 7 |
| *mKIAA0621* | 18 |
| *mKIAA4074* | 19 |
| *Mosc2* | 1 |
| *Mov10* | 3 |
| *Mxra8* * | 4 |
| *Myl4* | 11 |
| *Nipsnap3a* * | 4 |
| *Nrbp2* | 15 |
| *Nsd1* * | 13 |
| *Oprd1* | 4 |
| *Pcnxl3* | 19 |
| *Pdgfa* | 5 |
| ***Peg10*** | 6 |
| *Pip4k2b* | 11 |
| *Pklr* | 3 |
| *Ppm1j* | 3 |
| *Prhoxnb* | 5 |
| *Prosapip1* | 2 |
| *Rab44* | 17 |
| *Rhoc* | 3 |
| *Rnf214* | 9 |
| *Rps4x* | 6 |
| *Scn1b* | 7 |
| *Scn8a* | 15 |
| *Sec24c* | 14 |
| *Shank2* | 7 |
| *Sipa1* | 19 |
| *Slc26a10* | 10 |
| *Snx20* | 8 |
| *Socs5* | 17 |
| *Sorbs3* | 14 |
| *Stbd1* | 5 |
| *Svop* | 5 |
| *Tacc2* | 7 |
| *Tas1r3* | 4 |
| ***Th*** | 7 |
| *Tmem209* | 6 |
| *Tmem211* | 5 |
| *Tom1l2* | 11 |
| *Tpst1* | 5 |
| *Trerf1* | 17 |
| *Trim2* | 3 |
| *Tsc22d1* | 14 |
| *Vps37b* | 5 |
| *Wdr81* | 11 |
| ***Zrsr1*** | 11 |
| **Bold** = known imprinted gene  * = Confirmed by RNAseq | |
